# GATA6 coordinates cross-talk between BMP10 and oxidative stress axis in pulmonary arterial hypertension

**DOI:** 10.1038/s41598-023-33779-8

**Published:** 2023-04-22

**Authors:** Tetsuo Toyama, Tatiana V. Kudryashova, Asako Ichihara, Stefania Lenna, Agnieszka Looney, Yuanjun Shen, Lifeng Jiang, Leyla Teos, Theodore Avolio, Derek Lin, Ulas Kaplan, Grace Marden, Vrinda Dambal, Dmitry Goncharov, Horace Delisser, Robert Lafyatis, Francesca Seta, Elena A. Goncharova, Maria Trojanowska

**Affiliations:** 1 Arthritis and Autoimmune Diseases Center, Boston University School of Medicine, 75 E. Newton St. Evans Building, Boston, MA 02118 USA; 2 Pittsburgh Lung, Blood and Heart Vascular Medicine Institute, University of Pittsburgh School of Medicine, Pittsburgh, PA USA; 3 Division of Pulmonary, Allergy and Critical Care, University of Pittsburgh School of Medicine, Pittsburgh, PA USA; 4 Division of Pulmonary, Critical Care, and Sleep Medicine, Davis School of Medicine, University of California, Davis, CA USA; 5 Perelman School of Medicine, University of Pennsylvania, Philadelphia, PA USA; 6 Division of Rheumatology and Clinical Rheumatology, Department of Medicine, University of Pittsburgh, Pittsburgh, PA USA; 7 The Genome and Biomedical Science Facility (GBSF), Rm 6523, 451 Health Sciences Drive, Davis, CA 95616 USA

**Keywords:** Experimental models of disease, Preclinical research, Cell biology, Pathogenesis, Cardiovascular biology

## Abstract

Pulmonary arterial hypertension (PAH) is a life-threatening condition characterized by a progressive increase in pulmonary vascular resistance leading to right ventricular failure and often death. Here we report that deficiency of transcription factor GATA6 is a shared pathological feature of PA endothelial (PAEC) and smooth muscle cells (PASMC) in human PAH and experimental PH, which is responsible for maintenance of hyper-proliferative cellular phenotypes, pulmonary vascular remodeling and pulmonary hypertension. We further show that GATA6 acts as a transcription factor and direct positive regulator of anti-oxidant enzymes, and its deficiency in PAH/PH pulmonary vascular cells induces oxidative stress and mitochondrial dysfunction. We demonstrate that GATA6 is regulated by the BMP10/BMP receptors axis and its loss in PAECs and PASMC in PAH supports BMPR deficiency. In addition, we have established that GATA6-deficient PAEC, acting in a paracrine manner, increase proliferation and induce other pathological changes in PASMC, supporting the importance of GATA6 in pulmonary vascular cell communication. Treatment with dimethyl fumarate resolved oxidative stress and BMPR deficiency, reversed hemodynamic changes caused by endothelial Gata6 loss in mice, and inhibited proliferation and induced apoptosis in human PAH PASMC, strongly suggesting that targeting GATA6 deficiency may provide a therapeutic advance for patients with PAH.

## Introduction

Pulmonary Arterial Hypertension (PAH) is a life-threatening disease characterized by vasoconstriction and remodeling of pre-capillary pulmonary arterioles, leading to restriction of blood flow, increases in pulmonary arterial pressure and pulmonary vascular resistance, and eventually right ventricular failure and death^1,2^. Untreated, median survival is 2.8 years, and even with current treatment regimens, the prognosis remains poor (mortality rate of 10–15% yearly)^3–5^. The disease remains poorly managed especially in patients with rapid progression, and lung transplant is the only option for final-stage patients.

Pulmonary vascular remodeling is a complex process with multi-cellular involvement. In established PAH, enhanced growth and survival of PA smooth muscle cells (PASMC) and hyper-proliferation of endothelial cells (PAEC) significantly contribute to obliteration of the lumen of small PAs, pulmonary vascular remodeling and overall PAH^6–8^.

GATA6 is one of six highly conserved transcription GATA factors with two tandem zinc fingers that interact with other transcriptional regulators and bind the canonical DNA motif, (G/A)GATA(A/T)^9^. Human GATA6 is expressed in a wide array of tissues and maintains the differentiated cell phenotypes^10,11^. In vasculature, GATA6 has been primarily investigated in systemic VSMC. It is highly expressed in quiescent VSMC and is rapidly downregulated in injured vessels^12^. Rescuing Gata6 levels in balloon-injured carotid arteries resulted in VSMC differentiation and reduced neointimal formation in rats^12^. Global transcriptomic analysis identified GATA6 as a crucial regulator of VSMC fate in the arterial tree^13^, further supporting its importance in systemic vascular homeostasis.

Our knowledge about the role of GATA6 in pulmonary vasculature, however, is limited by the paper describing Gata6 loss in PAs from rats with pneumonectomy and monocrotaline (MCT)-induced PH^14^ and a more recent report from our group focused on endothelial GATA6 loss in PH development^15^. Our studies showed that GATA6 is deficient in both endothelium and VSMC in IPAH and SSc-PAH^15^. Notably, mice with conditional deletion of *Gata6* in endothelial cells (*Gata6*-CKO) develop PH spontaneously, suggesting that GATA6 downregulation is an early and key event that leads to endothelial dysfunction and the development of PH^15^. However, the mechanisms by which GATA6 loss induces endothelial injury and the subsequent development of PH has not been elucidated.

This study was undertaken to clarify the role and functional significance of GATA6 deficiency in pulmonary vasculature and to evaluate the potential of the GATA6 axis as a therapeutic target in PAH. Here we report on novel functions of GATA6 as a regulator of oxidative stress and BMP10 signaling. We also show that dimethyl fumarate (DMF) is very effective in alleviating the pathological changes induced by Gata6 loss in vivo.

## Methods

### Human tissues and cell cultures

All experimental procedures using human tissues or cells conformed to the principles outlined in the Declaration of Helsinki. De-identified lung tissue specimens from patients with PAH and non-diseased (control) donors were obtained from the University of Pittsburgh Tissue Medical Center Lung Transplant program and Tissue Donation Program at the University of Pittsburgh Medical Center. All experiments were performed in accordance with the protocols approved by the University of Pittsburgh Institutional Review Board (IRB) and Committee for Oversight of Research and Clinical Training Involving Decedents (CORID). Patient characteristics are included in Supplemental Table 4. Early-passage^3–8^ distal human PASMC and PAECs from non-diseased subjects or patients with PAH were provided by the University of Pittsburgh Cell Processing Core and Pulmonary Hypertension Breakthrough Initiative (PHBI) in accordance with PHBI, IRB, and CORID protocols. Human subjects’ characteristics are provided in the Supplemental Table 4. Prior tissue collection, an informed consent was obtained from all subjects and/or their legal guardian(s) by PHBI, University of Pittsburgh Medical Center Lung Transplant program and Tissue Donation Program in accordance with IRB and CORID policies. Cell isolation and maintenance were performed as described previously^16,17^. Control PAECs were purchased from Lonza (Walkersville, MD) and cultured in complete endothelial growth medium (EGM)-2 (Lonza) or endothelial cell basal medium MV2 (PromoCell, Heidelberg, Germany).

### Mice

All experimental procedures were performed in accordance with the protocol reviewed and approved by the Boston University Institutional Animal Care and Use Committee (protocol TR2190057). The investigation conformed to the Guide for the Care and Use of Laboratory Animals, published by the US National Institutes of Health (NIH). The study was carried out in compliance with the ARRIVE guidelines. Generation of Gata6 CKO mice has previously been described^15^. Briefly, Gata6 flox/flox mice were purchased from Jackson Laboratory (Bar Harbor, ME). The mouse line, which contains *loxP* elements in the introns flanking exon 2 of the *Gata6* gene, was bred with mice expressing the Cre recombinase under the control of the endothelial-specific VE-cadherin promoter (Jackson Laboratory). DMF (Sigma Aldrich) intraperitoneal injections were performed daily for three weeks with a dose of 90 mg/kg. DMF was dissolved in DMSO (30 mg/ml) and mixed with pre-warmed (37 °C) vehicle (10% (2-Hydroxypropyl)-β-cyclodextrin (Sigma Aldrich) in PBS) immediately prior to injection. The volume of a single injection was 500 µl. The control group was injected with equal volume of vehicle/DMSO solution. Six- to eight-weeks old Gata6 CKO and control littermate mice were used for the experiment. In our previous study^15^, we detected no significant differences in endothelial Gata6 signaling and PH severity between male and female Gata6 CKO mice. Because of this, mice of both sexes were used equally in all performed experiments as permitted by the sex of offsprings in the litter. The left lung was fixed by tracheal perfusion either with 4% paraformaldehyde or 50% OCT (Thermo Fisher Scientific) in PBS and right lung was used for RNA or protein isolation.

### Mouse endothelial cell isolation

Two different methods were used to isolate mouse endothelial cells (ECs). Mouse lung tissue was finely minced and digested with the Lung Dissociation Kit (MACS Miltenyl Biotec) using the MACS Dissociator technology according to the manufacturer’s protocol. Debris was removed by sequential filtration through 70 and 40 μm filters (BD Biosciences, San Jose, CA). For the isolation of fresh cells for mRNA analyses, cells were labeled with magnetic microbeads (CD31 and CD45) and CD31 + /CD45 − cells were collected using an autoMACS^®^ Pro Separator (Miltenyi Biotec). For the isolation of fresh cells for staining assay, cells were stained with fluorochrome conjugated mouse-specific antibodies, CD31-FITC and CD45-APC from BD Biosciences, and CD31 + /CD45 − cells were collected after cells were sorted by a MoFlo High Speed Cell Sorter (BD Biosciences).

### BMP9 and BMP10

BMP9 (GDF-2) and BMP10 were purchased from PeproTech (Cat.no 120–07 and 120–40, respectively). Recombinant Human BMP-10 is a 24.4 kDa homodimeric disulfide-linked protein consisting of two 108 amino acid subunits, which correspond to amino acid residues 317 to 424 of the full-length BMP-10 precursor. Recombinant Human GDF-2 is a 24.1 kDa disulfide-linked homodimeric protein consisting of two 110 amino acid polypeptide chains.

### Immunohistochemical analysis

4% paraformaldehyde-fixed, paraffin-embedded 5-μm sections after dewaxing and heat antigen retrieval in Tris/EDTA pH 9.0 for 20 min were used for staining. Blocking was achieved using 3% H2O2 followed by BLOXALL (Vector Labs, Burlingame, CA) or 2% horse or goat serum. Appropriate Vector HRP-ImmPress Polymers (mouse, rat, and rabbit) were used to detect primary antibodies, following fluorescent CF^®^ dye tyramide conjugates for tyramide signal amplification (Biotium, Fremont, CA): CF^®^488A and CF^®^594 tyramide according to the manufacturer’s instructions. Each labeling step was followed by HRP quenching with 3% H2O2 before the next stain. Typically, primary antibodies were re-titrated for use with tyramide amplification. Coverslips were mounted by using Vectashield with DAPI (Vector Laboratories). Immunofluorescence slides were imaged using an Olympus FlouView FV10i confocal microscope (Olympus, Waltham, MA) and Keyence BZ-X810 Fluorescent Microscope. Morphometrics were performed using the FIJI distribution of ImageJ. Images used in each morphometric analysis were captured at the same time using the same microscope and camera settings. Antibodies were as follows: rabbit anti-mouse/human GATA6 (Cell signaling technology, 58515 Clone D61E4, 1:2000), rat anti-mouse CD31 (Dianova DIA310 Clone SZ31, Hamburg, Germany, 1:2000), goat anti-/human alpha-SMA (Abcam, ab 21027,1:1500).

### RNA extraction, quantitative RT-PCR

RNA was isolated and purified from mouse total lungs, mouse lung ECs, human PAECs (HPAECs) and human VSMCs (HVSMCs) using the Qiagen RNeasy kit and RNA clean & concentrator (Zymo Research) following the manufacturer’s instructions. Total RNA from cultured cells was isolated using the TRI Reagent (MRC Inc.) The lung was ligated and saved in RNAlater (Life Technologies), homogenized with TissueLyser II (Qiagen) at 20 Hz for 1 min, and total RNA was isolated with the RNeasy Mini Kit (Qiagen). One μg of total RNA was reverse transcribed with random hexamers using the Transcriptor First Strand cDNA Synthesis kit (Roche Applied Science) according to the manufacturer's protocol. Diluted cDNA was mixed with SYBR^®^ Green PCR Master Mix (Applied Biosystems) to quantitatively measure gene expression using a StepOnePlus Real-Time PCR system (Applied Biosystems). Relative change in the levels of genes of interest was determined by the 2−ΔΔCT method using house keeping genes. Expression of the housekeeping genes GADPH (for HPAECs and HVSMCs) and b2MG (mouse lungs) served as internal references in each assay performed. The mouse primers are listed in Supplemental Table 5 and human primers in Supplemental Table 6.

### Microarray analysis

Microarray analysis was performed at The Boston University Microarray Core Facility. Affymetrix CEL files were normalized to produce gene-level expression values using the implementation of the Robust Multiarray Average (RMA) in the affy package (version 1.36.1) included within in the Bioconductor software suite (version 2.12) and an Entrez Gene-specific probe set mapping from BrainArray (version 16.0.0). RLE and NUSE quality metrics were computed using the affyPLM Bioconductor package (version 1.34.0). All microarray analyses were performed using the R environment for statistical computing (version 2.15.1).

### Mitochondrial function assessment

The oxygen consumption rate (OCR) was measured using the Seahorse XFe96 analyzer (Seahorse Bioscience) following the manufacturer’s instructions. HPAECs were seeded on collagen-coated XFe96 microplates at the concentration of 20,000 cells per well 48 h before the assay. HPAECs were transfected with siRNA against GATA6 or non-silencing siRNA 24 h before the Seahorse analysis. HPAECs were maintained in assay medium in a non-CO_2_ incubator for 45 min before the assay. Proton leak was measured in the presence of oligomycin (2 mM) (Sigma Aldrich), maximum respiration was evaluated in the presence of carbonyl cyanide-4-(trifluoromethoxy) phenylhydrazone (FCCP; 2.5 mM) (Sigma Aldrich), which uncouples ATP synthase and the electric transport chain, and spare respiratory capacity was evaluated in the presence of the electric transport chain inhibitors antimycin A (4 mM) (Sigma Aldrich).

### RNA-sequencing, clusters and pathway analysis

RNA-seq analysis was performed on human PAH PASMC from three different subjects that were infected with AdGATA6 or mock-infected for 48 h. RNA-seq data was normalized by Transcripts Per Million (TPM), and clustered (Cluster 3.0) using Euclidean distance and complete linkage (see Supplemental Table 2 for complete gene expression data). Genes in clusters up- and down-regulated were analyzed for Gene Ontology pathways. Please refer to supplemental Tables 2 and 3 for complete dataset and up- and down-regulated pathways analysis.

### Antioxidant enzyme activity measurements

Antioxidant activities of superoxide dismutase (SOD), glutathione peroxidase (GPX) and catalase were measured by spectrophotometric assays using commercial kits (Cayman). These methods assess the functional capacity of the enzymes to act on their substrates. Total SOD activity was measured using a tetrazolium salt for detection of superoxide radicals generated by xanthine oxidase and hypoxanthine. GPX activity was indirectly determined by coupled reaction with glutathione reductase. The activities are given in nmol/min/mL and all determinations were run in triplicates.

### Adenoviral constructs

Adenoviral vectors expressing GATA6 and green fluorescent protein (GFP) and adenovirus expressing GFP alone (AdGFP) as a control vector were generated as previously described^18^. Ad-CMV-GATA6 and Ad-CMV-eGFP-GATA6 were purchased from Vector Biolabs. The dose used to transduce cells was 20 multiplicities of infection of the adenovirus (MOI). Lentiviral vectors producing shRNA GATA6 (shGATA6) or scrambled control shRNA (shCtrl) were purchased from Santa Cruz Biotechnology (Santa Cruz, CA). RNA, immunoblot analyses and functional assays were performed 48 or 72 h after infection.

### Reactive oxygen species (ROS) detection

HPAECs treated with GATA6 siRNA or SCR siRNA, ECs isolated from Gata6 CKO or WT mouse lungs, or human non-diseased PASMC infected with shGATA6 or shCtrl, grown on coverslips (HPAECs and ECs) or chamber slides (PASMC), were stained with CellROX, MitoSOX and NucBlue (Life Technologies) according to manufacturer protocol. The ROS levels were determined using fluorogenic dyes for selective detection of superoxide in the mitochondria (MitoSOX™ Red mitochondrial superoxide indicator) and general oxidative stress (CellROX green) in live cells following the manufacturer’s instructions. Briefly, cells were incubated in media containing MitoSOX (5 µM) and CellRox (5 µM) for 30 min at 37 °C in the dark, washed with PBS and imaged using fluorescence microscopy. Endothelial cells were categorized based on the signal positivity. In PASMC, the intensity of the CellROX or MitoSOX signal was measured. Analysis was performed using ImageJ software.

### siRNA transient transfection

Eighty percent confluent cultures of HPAECs or PASMC were transfected with 10 nM of small interfering RNA (siRNA) directed against GATA6 (ON-TARGET plus SMART pool™, Dharmacon, Waltham, MA) and control siRNA using Invitrogen™ Lipofectamine™ RNAiMAX Transfection Reagent (Invitrogen). Forty-eight to seventy-two hours post-transfection, total RNA was prepared using TRI reagent (MRC, Inc., Cincinnati, OH) according to the manufacturer’s protocol, whole cell lysates were prepared for immunoblot analysis, or functional assays were performed.

### Immunoblot analysis

Cells were collected and washed with PBS. Cell pellets were lysed in radioimmunoprecipitation assay buffer (Thermo Fisher scientific) or buffer (described previously in^16,17^ with freshly added phosphatase inhibitor and protease inhibitor mixture (Sigma-Aldrich). Pieces of lung tissue were homogenized with Tissue Lyser ll (Qiagen) and lysed in the same buffer as cells. Protein concentration was quantified using the BCA Protein Assay kit (Pierce, Waltham, MA). Equal amounts of total protein per sample were separated via SDS–polyacrylamide gel electrophoresis and transferred to nitrocellulose or PVDF membranes (Bio-Rad). The membranes were blocked with nonfat dry milk in TBST and then probed overnight with appropriate primary antibodies. After TBST washes, membranes were probed with horseradish peroxidase-conjugated secondary antibodies against the appropriate species for one hour at room temperature and developed using the Chemiluminescent detection kit (Pierce). For next antibody probing, membranes were stripped with stripping buffer (Thermo Fisher Scientific) and reprobed. Band intensities were determined by densitometric analysis with ImageJ software. Antibodies were as follows: Goat anti-human GATA6 (R&D; AF1700, 1:500), mouse anti-human GATA6 (Santa-Cruz, 517554, 1:500), Rabbit anti-human/mouse SOD2 (Cell signaling technology; 13141, 1:1000; 13145, 1:5000), Goat anti-human ALK1 (R&D; AF370, 1:500), Rabbit anti-mouse ALK1 (ABGENT AP7807a, 1:1000), rabbit anti-human/mouse ActR2B (LS bio; LS-B7781, 1:1000), mouse anti-human BMPR2 (Novus; NBP2-37624 Clone 3F6; 1:1000), rabbit anti-mouse BMPR2 (Proteintech; 19087–1-AP; 1:1000), mouse anti-mouse/human/rat BMPR2 (Abcam, catalog #130206 1:500), rabbit anti-human/mouse Endoglin (Proteintech; 10862–1-AP; 1:1000), rabbit anti-mouse BiP (Abcam; ab53068; 1:1000), rabbit anti-mouse CHOP (Novus; NBP2-13172; 1:1000), mouse anti-beta-actin (Sigma; A1978; 1:2000), mouse anti-human PRPF4 (Abcam, 69878, 1:1000), rabbit anti-human MYEOV (Invitrogen, PA5-48938 1:1000), rabbit anti-human STING (Invitrogen, PA5-26751, 1:1000), rabbit anti-human/mouse/rat EAF1 (Invitrogen, PA5-100493, 1:1000), rabbit anti-human/mouse/rat Histone H3 (Cell Signaling, 9715 1:1000), rabbit anti-human/mouse/rat a-b Tubulin (Cell Signaling, 2148, 1:1000). Immunoblot acquisition and analysis was performed using ImageJ (NIH, Bethesda, MD) in compliance with the digital image and integrity policies.

### Chromatin immunoprecipitation assay

Chromatin immunoprecipitation was performed as previously described using ChIP grade GATA6 antibody (Cell signaling technology 58515 Clone D61E4). Real time qPCR was performed in triplicate using specific primers for the human *SOD2, GPX1, GPX7, BMPR2, ALK1 and ActR2B*, followed by agarose gel electrophoresis. (Supplemental Table 7). Open reading frame (ORF) primers were used as a negative control. The amount of immunoprecipitated DNA (percent recovery) was calculated from a standard curve generated with the serial dilutions of input chromatin.

### Echocardiography and measurement of RV pressure

Transthoracic echocardiography was performed using the Vevo 770 High-Resolution Imaging System with 30-MHz RMV-707b scanning head (VisualSonics, Toronto, Canada). Pulmonary acceleration time (PAT) and PAT as a fraction of ejection time were measured from the pulse-wave Doppler recordings of the PA blood flow. For assessment of right ventricular hypertrophy, the ventricles were excised and weighed. The weight ratio of the right ventricle to the left ventricle plus septum was calculated as an index of RV hypertrophy. Right ventricular pressures were measured with a high-fidelity pressure catheter inserted in the right ventricle via cannulation of the right jugular vein, to monitor the development of pulmonary hypertension. Briefly, mice were anaesthetized with an intraperitoneal injection of 100 µl of Ketamine (75 mg/kg) and Xylazine (10 mg/kg) and kept recumbent on a heating pad at 39 °C, to maintain body temperature during the surgical procedure. A 1.5 cm incision was performed vertically in the central neck area. The right jugular vein was gently isolated, free of connective tissue, and blood flow temporarily occluded with a 6–0 silk suture. A 1.2F Mikro-tip^®^ pressure catheter transducer (Model SPR-1000, Millar Instruments) was inserted in the jugular vein by puncturing the vein with a 27 G needle bent at 90°, and gently advanced into the right ventricle. Pressure waveforms were monitored in real time with the PowerLab Chart 8 data acquisition system (ADInstruments) and 10 min of stable pressure recordings were acquired for each mouse. RV pressures were calculated by averaging at least 60 cardiac cycles for each mouse.

### Nuclear fractioning

Nuclear fractions were prepared using the REAP cell fractionation method as described in^19^ with the following modification: the sonication step was substituted with shearing DNA by passing it through the insulin syringe 5–10 times.

**Cell counts, DNA synthesis (BrdU incorporation), proliferation (Ki67), apoptosis (Annexin V), and immunocytochemical analyses** were performed as we described previously^16,17,20,21^. Cell counts were performed using Countess II FL Automated Cell Counter (Thermo Fisher Scientific, Waltham, MA) according to the manufacturer’s protocol. Briefly, equal number of cells per well was plated in a six-well plate, cells were serum-deprived for 48 h, stained with trypan blue, and subjected to automatic cell counts (3 technical replicates per well). Ki67 assay was performed as follows: cells were washed with phosphate-buffered saline (PBS), fixed in 4% paraformaldehyde/PBS, permeabilized with Triton X-100/PBS, and blocked in 2% BSA/PBS, followed by immunocytochemical analysis with primary anti-Ki-67 antibody (#9129, Cell Signaling, Danvers, MA) and secondary chicken anti-rabbit IgG (H + L) Alexa Fluor^®^ 594 antibody (#A-21442, Invitrogen, Waltham, MA). 4′,6-diamidino-2-phenylindole (DAPI) staining was performed to detect nuclei. Images were taken using a Keyence BZ-X800 microscope. Apoptosis analysis was performed using Annexin V-FITC Apoptosis Staining/Detection Kit (ab14085, Abcam) following the manufacturer’s protocol with modifications for microplate reader. Briefly, an equal number of human PASMC or PAEC were plated in each well of 96-well plate (100 000 cells/well). 48 h later, cells were transfected with mock ( − ) or AdGATA6 for 48 h, and Annexin V-FITC apoptosis staining procedure for adherent cells was performed.

### Statistics

Analyses were performed using Prism 8 (GraphPad) and StatView software. Values are presented as means ± SE. Normality was assessed using Kolmogorov–Smirnov test. Statistical analysis of two groups of independent samples with sample sizes of less than six per group or larger than six but failing a test of normality was performed using non-parametric Mann–Whitney U test. Comparison of three or more independent groups with sample sizes of less than six per group or larger than six but failing a test of normality was performed using non-parametric Kruskal–Wallis test. If there was a significant difference among groups, the post hoc Dunn’s test for multiple comparisons was performed. For comparison of two groups of independent samples with sample sizes of larger than six per group that passed normality test, unpaired t test was used. To compare three or more independent groups with normal distribution and n ≥ 6/group, one-way ANOVA followed by post hoc Tukey’s multiple comparisons test was used. Correlations are Spearman analyses with Holm–Sidak adjusted p values. In all cases, p < 0.05 were considered significant and are abbreviated in the figures as follows: *p < 0.05, **p < 0.01, ***p < 0.001 and ****p < 0.0001.

## Results

### GATA6 deficiency induces oxidative stress and mitochondrial dysfunction by suppressing anti-oxidant enzymes in human PAEC (HPAEC) and Gata6 CKO mice

**Figure 1:**
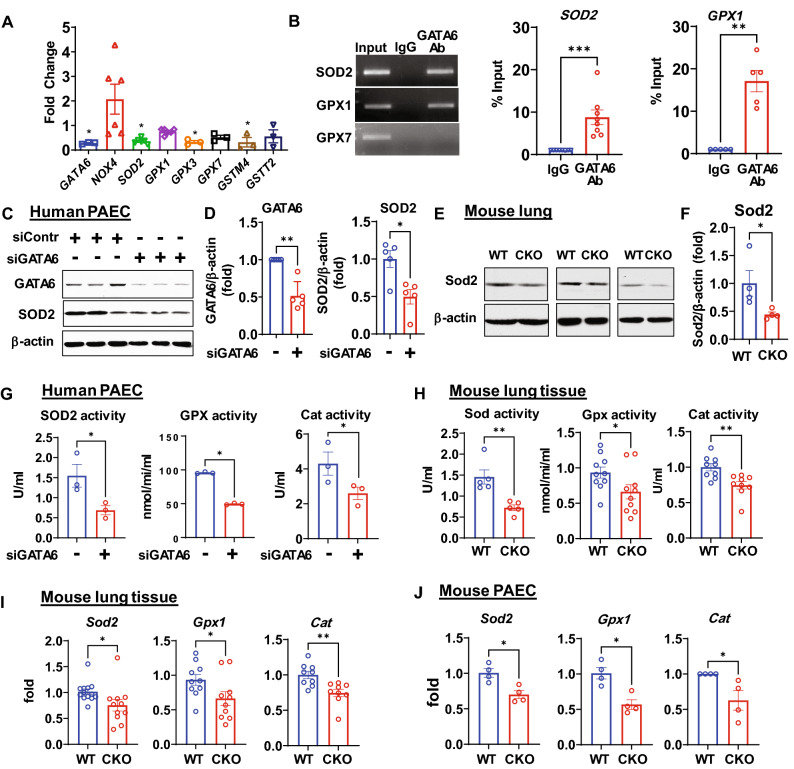
GATA6 loss induces SOD2 and other anti-oxidant enzymes deficiency in human and mouse PAECs. (**A**) An expression of the antioxidant enzymes measured by qPCR in human PAECs (HPAECs) transfected with siGATA6 or control scr siRNA. mRNA levels in scr siRNA transfected cells were set as one. Data are means ± SE, *p < 0.05 vs scr siRNA by Kruskal–Wallis test with post hoc Dunn’s test for multiple comparisons; n = 3–10 (see also Supplemental Table 1). (**B**) Chromatin immune precipitation (ChiP) assay in HPAECs, n = 8 for *SOD2*; n = 5 for *GPX1*. The experiments were repeated three times. Representative gel images and data quantification are shown. Values are means ± SE, **p < 0.01, ***p < 0.001 by Mann Whitney U test. (**C,D**) Control HPAECs transfected with siGATA6 or scr siRNA (siContr) were assayed by Western blot analysis to detect GATA6 and SOD2. Representative blots are shown. Values are means ± SE of the relative protein levels by densitometry, n = 5, **p < 0.01 by Mann Whitney U test. (**E,F**) Western blot analysis of whole lung tissue from Gata6 CKO and WT mice. Representative blots are shown. Values are means ± SE of the relative protein levels by densitometry, n = 4, *p < 0.05 by Mann Whitney U test. (**G**) SOD2, GPX, and Catalase activity were measured in Human HPAECs transfected with siGATA6 or scr siRNA, Values are means ± SE, n = 3, *p < 0.05 by Mann Whitney U test. (**H**) Mouse Sod2 (n = 5), Gpx (n = 10), and Cat (n = 9) activity were measured in mouse lung tissue in Gata6 CKO and WT mice. *p < 0.05, **p < 0.01 by Mann Whitney U test (Sod activity) and unpaired τ test (Gpx and Cat activities). (**I,J**) mRNA levels of the antioxidant enzymes measured by qPCR in whole lung tissues (**I**) and in PAEC from Gata6 CKO mice (J). Data are means ± SE. I: n = 12–13 for *Sod*, n = 10/group for *Gpx1*, n = 9/group for *Cat*. J: n = 4 mice/group. *p < 0.05, **p < 0.01 by unpaired τ test (I) or Mann Whitney U test (**J**). The original blots are presented in Supplementary Fig. S13.

The mechanisms by which GATA6 deficiency contributes to PAH development are not well understood. To determine the effects of GATA6 deficiency on human PAEC (HPAEC), we performed transcriptome analysis on HPAEC transfected with scrambled or GATA6 siRNAs (Table S1). The data revealed downregulation of multiple genes involved in the anti-oxidant stress response, including *SOD2*, and several glutathione peroxidase (*GPX*) family members, but not NOX enzymes (Fig. 1A) or nuclear factor erythroid 2-related factor 2 (*NRF2*) (data not shown). Similar changes were also observed in PAEC from mice with endothelial-specific Gata6 loss (Gata6 CKO) compared to controls (Fig. S1). Chromatin immunoprecipitation analysis indicated that GATA6 acts as a direct transcriptional regulator for selected antioxidant genes such as *SOD2* and *GPX1* (Fig. 1B).

Consistent with the transcriptome analysis, protein levels of SOD2 were decreased in both HPAEC with siRNA-induced GATA6 deficiency and Gata6 CKO mouse lung (Fig. 1C–F). Further supporting this data, siRNA GATA6-transfected HPAEC and Gata6 CKO mouse lung showed significantly lower enzyme activities of SOD2, GPX, and catalase compared to Scr siRNA-transfected cells ( − ) and wild type (WT) mouse lung (Fig. 1G,H). mRNA levels of *Sod2, Gpx1*, and catalase were also significantly decreased in the lung tissues and PAEC from the Gata6 CKO mice (Fig. 1I,J). These data suggest that GATA6 is a novel transcription activator of the anti-oxidant enzymes in PAEC.

Lower levels of SOD2 and other anti-oxidant enzymes in GATA6-deficient PAEC have suggested deregulation of ROS production. To monitor mitochondrial superoxide levels in intact cells, we employed a mitochondria specific fluorescent probe MitoSOX. In addition, ROS production was measured at the same time by the CellROX Deep Red Reagent fluorogenic probe. Both, cellular ROS and mitochondrial superoxide were significantly elevated in GATA6-deficient HPAEC and PAEC obtained from Gata6 CKO mice (Supplemental Fig. S2A–D).

We next measured the effects of GATA6 deficiency on mitochondrial respiration using the Seahorse extracellular flux analyzer (Supplemental Fig. S3). HPAEC with siRNA-induced GATA6 deficiency showed significantly higher mitochondrial membrane potential, compromised basal and maximal respiration, as well as slightly lower ATP production suggesting mitochondrial impairment. Additionally, GATA6-deficient HPAEC had abnormally reduced proton leak, which may contribute to the elevated ROS production. Together, these data suggest that endothelial deficiency of GATA6 induces oxidative stress and alters mitochondrial function, which may contribute to PAH development.

### GATA6 is deficient in PAEC and PASMC from human PAH lungs

**Figure 2:**
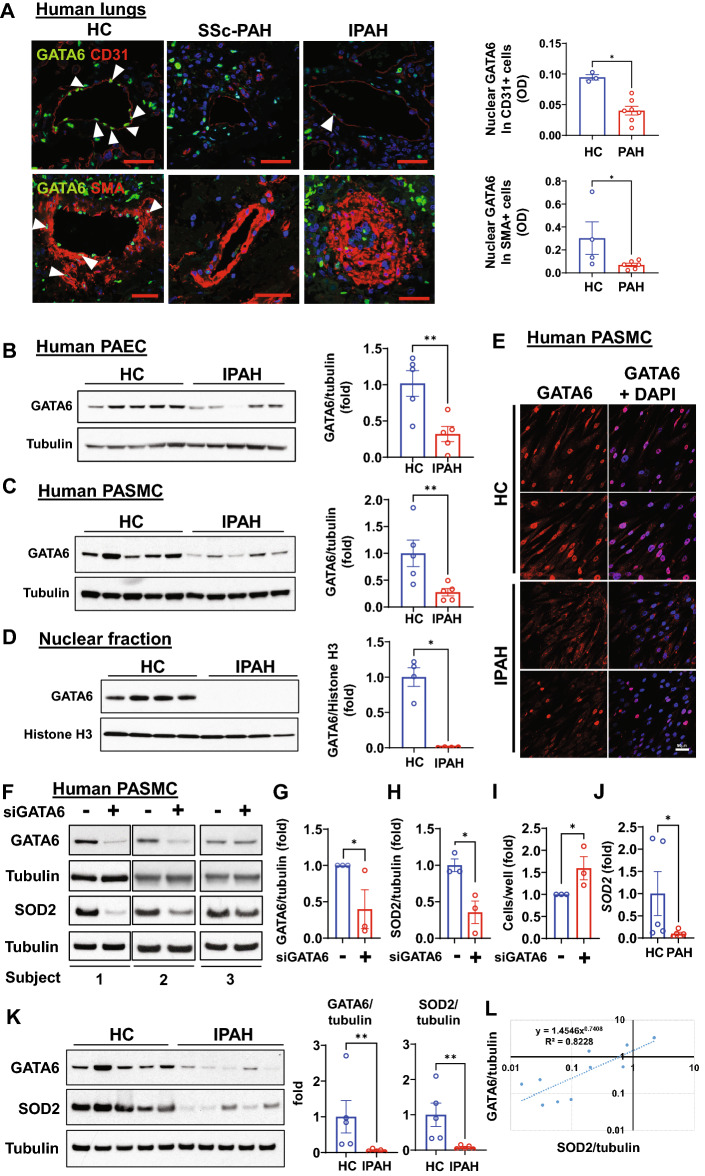
GATA6 is deficient in both PAEC and PASMC in human PAH lungs. (**A**) IHC analyses were performed to detect GATA6 (green), CD31 (red) and α-SMA (red) in small PAs from healthy controls (HC) and patients with PAH (SSc-PAH and IPAH) and analysis of nuclear GATA6 in CD31- and SMA-positive cells in small PAs was performed. Left: Images are representative from 3–4 subjects/control, SSc-PAH, and IPAH. Bar equals 50 μm. White arrowheads indicate GATA6-positive cells. Right: Data are in optical density units (OD); Data are means ± SE from 3–4 human subjects per control, 7–8 for PAH (SSc-PAH + IPAH) groups. *p < 0.05 by Mann Whitney U test. (**B,C**) Immunoblot analysis of early-passage PAEC and PASMC from healthy controls (HC) and subjects with IPAH to detect indicated proteins. Data are means ± SE from n = 5 subjects/group. **p < 0.01 by Mann Whitney U test. (**D**) Immunoblot analysis of nuclear fractions of PASMC from healthy control (HC) and IPAH subjects. Data are means ± SE from n = 4 subjects/group. *p < 0.05 by Mann Whitney U test. (**E**) Immunocytochemical analysis of PASMC from healthy control (HC) and IPAH subjects to detect GATA6 (red) and DAPI (blue). Bar equals 50 µm. Representative images from two subjects/group by Mann Whitney U test. (**F–J**) Human non-diseased PASMC were transfected with siRNA GATA6 or control scrambled siRNA ( − ). 48 h post-transfection, immunoblot (F, G) and cell count analysis (H) were performed. Data are means ± SE from n = 3 subjects/group. *p < 0.05 by Mann Whitney U test. (**I**) qPCR analysis of PASMC from healthy controls (HC) and patients with PAH to detect *SOD2* expression. Data are means ± SE, n = 5 subjects/group. *p < 0.05 by Mann Whitney U test. (**K,L**) Immunoblot analysis of healthy control (HC) and IPAH PASMC to detect indicated proteins was performed on the same membrane using strip- re-probe approach. Correlation between GATA6 and SOD was examined (R2). Data are means ± SE, n = 5 subjects/group. **p < 0.01 by Mann Whitney U test (**K**), R2 = 0.81 by Spearman analysis with Holm–Sidak adjusted p values (**L**). The original blots are presented in Supplementary Fig. S14.

We have previously reported that GATA6 is downregulated in both PAEC and PASMC in established PAH^15^. Previous studies of bulk RNA-sequencing are also consistent with a decline in GATA6 expression in IPAH, showing GATA6 expression in IPAH lungs is 0.86-fold expression in control lungs (adjusted p-value = 0.0214)^22^. Supporting previous observations, immunohistochemical and immunoblot analysis demonstrated reduced levels of GATA6 in both PAEC and PASMC in small remodeled PAs from patients with PAH (Fig. 2A) and in early-passage distal human IPAH PAEC and PASMC (Fig. 2B,C). Furthermore, in contrast to control PASMC, GATA6 was almost completely absent in nuclei from IPAH PASMC (Fig. 2D,E). Of note, SSc-PAH PAEC and PASMC were not available to perform the corresponding analyses.

To test whether GATA6 regulates SOD2 expression in PASMC, we directly depleted GATA6 in non-diseased PASMC using specific siRNA and showed that it resulted in reduced levels of SOD2 and increased cell growth (Fig. 2F–J). Similar to endothelial cells, GATA6 depletion in non-diseased (control) PASMC also resulted in significant increase of mitochondrial ROS and cellular ROS levels, as evidenced by higher MitoSOX and CellROX signal in shGATA6-infected PASMC compared to shContr-expressing cells (Supplemental Fig. S2E,F). Further supporting the importance of this signaling in human disease, there was a strong linear correlation between GATA6 and SOD2 protein levels in human PASMC, and both GATA6 and SOD2 were down-regulated in PASMC from human PAH lungs (Fig. 2K,L). Thus, correlation between SOD2 and GATA6 is consistent in both PAEC and PASMC.

### Restoration of GATA6 levels inhibits hyper-proliferation of pulmonary vascular cells from PAH lungs

**Figure 3:**
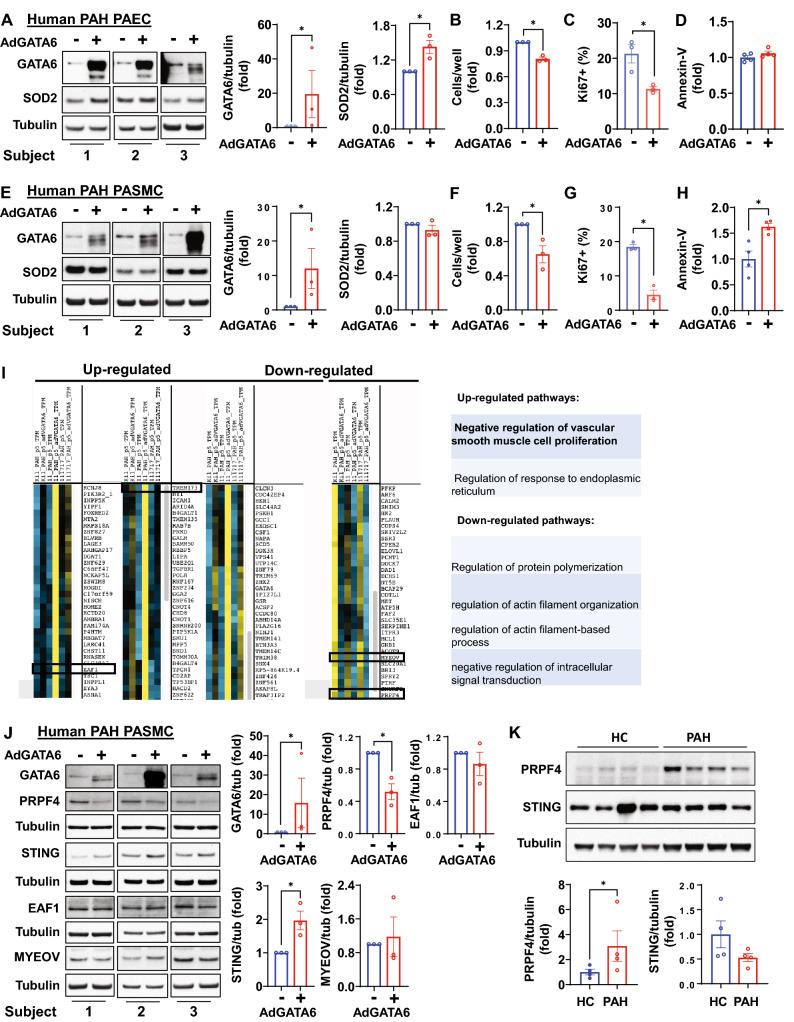
GATA6 deficiency is required for increased proliferation of human PAH PAEC and PASMC. (**A-D**) Human PAH PAECs were transfected with mock ( − ) (**A,B**), 10 MOI control adenovirus (**C,D**), or 10 MOI AdGATA6 for 48 h followed by immunoblot analysis (**A**), cell counts (**B**), proliferation (Ki67) (**C**), and apoptosis (Annexin V-FITC Apoptosis Staining/Detection Kit) (**D**) assays. Data are means ± SE from three (**A–C**) or four (**D**) experiments, each performed on the cells from different patient. *p < 0.05 by Mann Whitney U test. (**E–H**) Immunoblot analysis (**E**), cell counts (**F**), proliferation (Ki67) (**G**), and apoptosis (Annexin V-FITC Apoptosis Staining/Detection Kit) (**H**) assays of human PAH PASMC transfected with mock ( − ) (**E,F**), 10 MOI control adenovirus (**G,H**), or 10 MOI AdGATA6 for 48 h. Data are means ± SE from three (**E–G**) or four (H) experiments, each performed on the cells from different patient. *p < 0.05 by Mann Whitney U test. (**I**) RNA-sequencing analysis of adGATA6 and mock-transfected PAH PASMC (3 patients/group). RNA-sequencing data was normalized and clustered. Selected up and down-regulated gene clusters are shown. Genes further studied by immunoblot are boxed. Selected up- and down-regulated pathways identified by Gene Ontology analysis are shown (see supplemental Tables S2 and S3 for complete gene expression data). (**J**) Human PAH PASMC were transfected with mock ( − ) or 10 MOI AdGATA6 for 48 h followed by immunoblot analysis to detect indicated proteins. Data are means ± SE from three experiments, each performed on the cells from different patient. *p < 0.05 by Mann Whitney U test. (**K**) Immunoblot analysis of human healthy control (HC) and PAH PASMC to detect indicated proteins. Data are means ± SE from n = 4 subjects/group, *p < 0.05 by Mann Whitney U test. The original blots are presented in Supplementary Fig. S15.

To test the translational potential of targeting the GATA6 axis to mitigate hyper-proliferation of pulmonary vascular cells from PAH lungs, we transduced human PAH PAEC and PASMC with adenovirus encoding the human *GATA6* gene^18^. GATA6 re-expression normalized SOD2 protein levels and significantly reduced proliferation of PAH PAEC without significant effects on apoptosis (Fig. 3A–D, Fig. S4A). Surprisingly, re-expression of GATA6 in human PAH PASMC, while significantly reducing cell proliferation and inducing apoptosis, was unable to increase SOD2 levels (Fig. 3E–H, Fig. S4B), suggesting involvement of other downstream mechanisms. To gain insights into the mechanisms of GATA6 anti-proliferative action in human PAH PASMC, we re-expressed GATA6 in human PAH PASMC and performed RNAseq analysis followed by most up- and down-regulated pathway analysis. We found that GATA6 expression in PAH PASMC led to up- and down-regulation of multiple transcripts with the most profound up-regulation of transcripts belonging to the negative regulation of vascular smooth muscle cell proliferation (Fig. 3I, Tables S2, S3). Performing further validation of the RNAseq data, we selected two genes that are down-regulated by GATA6 expression (pro-proliferative *MYEOV* and *PRPF4)* and two growth suppressor genes that had been up-regulated by GATA6 (*TMEM173* and *EAF1)* (Fig. 3I) and performed immunoblot analysis. We found that GATA6 expression in PAH PASMC significantly decreased protein levels of pro-proliferative PRPF4 and elevated protein levels of growth suppressor STING (encoded by *TMEM173*) (Fig. 3J). Comparative analysis demonstrated that protein levels of PRPF4 were increased, while protein levels of STING were reduced in PAH PASMC compared to controls (Fig. 3K). Together, these data show that normalization of GATA6 levels in PAH pulmonary vascular cells reduces cell proliferation and implicates STING and PRPF4 in mediating the effects of GATA6 in PAH PASMC.

### BMP10 stimulates GATA6 expression through the ALK1/BMPR2/ENG/ERK1/2 pathway

**Figure 4:**
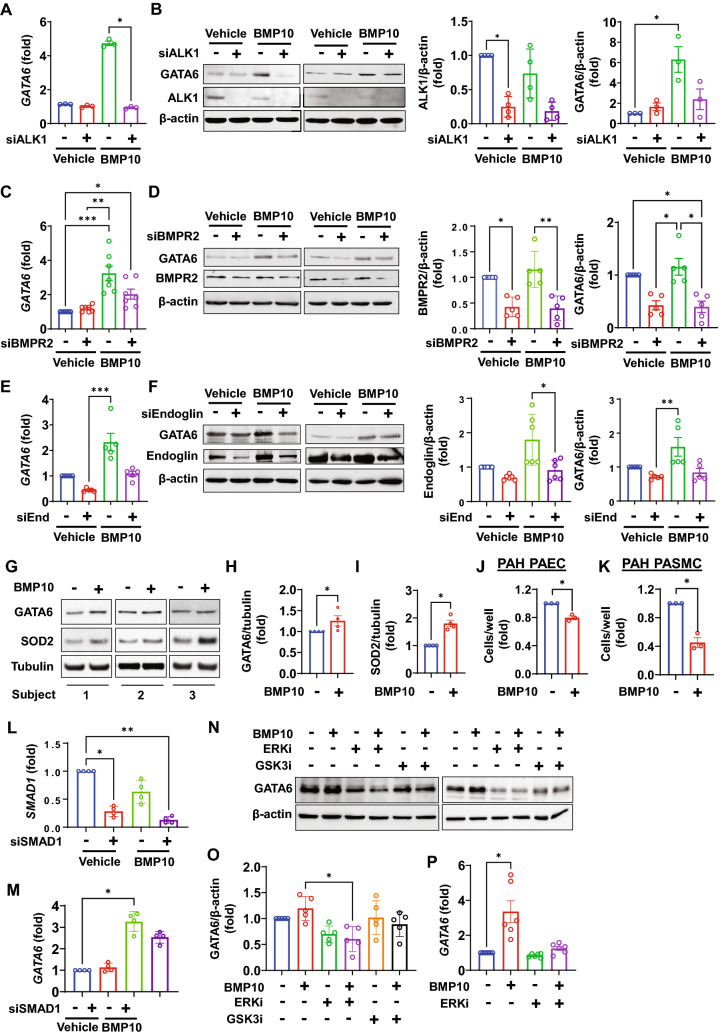
GATA6 expression is induced via BMP10-BMPR2/ALK1 axis. (**A–F**) HPAECs were transfected with ALK1, BMPR2, Endoglin, or control scr siRNA for 48 h, and then treated with 10 ng/ml BMP10 or vehicle for 6 h for RNA isolation and 24 h for protein isolation. (**A,C,E**): *GATA6* mRNA measured by qPCR. Data are means ± SE; each experiment was repeated at least three times. (**B,D,F**): GATA6 protein levels were measured by immunoblot analysis. Data are means ± SE, each experiment was repeated at least three times. Representative blots are shown. *p < 0.05, **p < 0.01, ***p < 0.001 by Kruskal–Wallis test with post hoc Dunn’s test for multiple comparisons. (**G–I**) Human PAH PASMC were treated with 10 ng/ml BMP10 or vehicle ( − ) for 48 h and immunoblot analysis to detect indicated proteins was performed. Data are means ± SE from n = 4 subjects/group. *p < 0.05 by Mann Whitney U test. (**J,K**) Equal amounts of human PAH HPAEC and PASMC were plated at 6-well plates and treated with 10 ng/ml BMP10 or vehicle ( − ). 48 h later cell counts were performed. Data are means ± SE from n = 3 subjects/group, 3 technical repetitions/subject. *p < 0.05 by Mann Whitney U test. (**L,M**) HPAECs were transfected with SMAD1 siRNA, and then treated with BMP10 for 6 h. *GATA6* and *SMAD1* mRNA levels were measured by qPCR. Data shown as means ± SE. Each experiment was repeated at least three times. *p < 0.05, **p < 0.01 by Kruskal–Wallis test with post-hoc correction for multiple comparisons. (**N,O**) HPAEC were treated for 30 min with diluent ( − ), 5 µM ERK1/2 inhibitor SCH772984 (ERKi), or 5 µM GSK3 inhibitor CHIR99021 (GSK3i) and then stimulated with BMP10 (10 ng/ml) or vehicle for 24 h. Representative immunoblots (**N**) and statistical analysis (**O**) are shown. (**O**): Data represent GATA6/β-actin ratio. Data are means ± SE from five independent experiments. *p < 0.05 by Kruskal–Wallis test with post-hoc Dunn’s correction for multiple comparisons. (**E**) HPAECs were treated with BMP10 in the presence or absence of 10 µM ERK1/2 inhibitor SCH772984 for 24 h. *GATA6* mRNA levels were measured by qPCR. Data shown as means ± SE. Each experiment was repeated six times. *p < 0.05 by Kruskal–Wallis test with post-hoc Dunn’s correction for multiple comparisons. The original blots are presented in Supplementary Fig. S16.

Upstream regulators of GATA6 are not well studied and the mechanisms of GATA6 deficiency in PAH remain to be established. BMP9 and BMP10 are central regulators of vascular homeostasis^23,24^. In adults, BMP9 is synthesized in the liver by hepatic stellate cells, while BMP10 is primarily synthesized in the right atrium, with lower expression also detected in the liver^25^. BMP9 and -10 are synthesized as preproproteins and are processed by furin-like convertase upon secretion. Biologically active BMP9 and -10 retain their respective pro-domains and are secreted into circulation as homo- and hetero-dimers^25^. BMP9 and BMP10 signal through the type I receptor ALK1, type II receptors Bmpr2 and ActRIIa or -b, and a co-receptor Endoglin^26^. The *BMP10* mutations were recently identified in young patients with severe PAH^27,28^, and mutations in *GDF2*, a gene encoding BMP9, resulting in BMP9 loss of function, result in reduced circulating levels of both, BMP9 and BMP10^29^. Mutational or epigenetic down-regulation at the receptor (predominantly BMPR2) level is strongly associated with familial and idiopathic PAH^30^. Importantly, BMPR2 down-regulation contributes to hyper-proliferative, apoptosis resistant phenotype in PAH pulmonary vascular cells^31–34^, and BMP9 is deficient in portopulmonary hypertension and is a biomarker for transplant-free survival^35^. Further supporting the link between BMP10 and pulmonary vascular disease, reduced circulating BMP10 and BMP9 have been associated with pulmonary vascular syndromes in patients with cirrhosis^36^. Given the central role of the BMPR2 signaling in PAH, we wished to investigate the potential interactions between the BMP9/10 and the GATA6 signaling pathway. We first assessed the effect of stimulation with mature forms of BMP9 or − 10 on GATA6 expression levels in HPAEC. BMP9 and − 10 similarly induced GATA6 expression (Fig. 4, Fig. S5), in agreement with a recent study of Salmon et al.^37^ that showed no difference in global gene expression between pro-BMP9 and pro-BMP10. Pro-BMP9/10 signal with similar potency as the mature, fully processed growth factor (GF) domains^37^. Further corroborating our results, GATA6 was among the top BMP9/10 upregulated genes^37^. The role of each BMP isoform in PAH is presently unclear, and the studies in animal models produced conflicting results^38^. In humans, PH is associated with increased right atrial stretch and pressure^39^, which may affect synthesis or release of BMP10. Whether BMP10 plays a more central role in PH than BMP9, is currently unknown. Given these recent findings and a less studied role of BMP10 in pulmonary vascular cells, we have selected BMP10 for the subsequent in vitro experiments.

BMP10 increased *GATA6* mRNA (Fig. 4A,C,E) and protein levels (Fig. 4B,D,F), while depletion of ALK1 (Fig. 4A,B), BMPR2 (Fig. 4C,D), or, to a lesser extent, endoglin (Fig. 4E,F) reduced this upregulation. Depletion of ActRIIA produced inconsistent results (data not shown). BMP10 also induced GATA6 and downstream SOD2 expression in PAH PASMC (Fig. 4G–I), suggesting that similar mechanisms are shared. Furthermore, treatment with BMP10 reduced proliferation of both PAH HPAEC and PAH PASMC (Fig. 4J,K). These data suggest that insufficient BMP9/10-BMP receptors signaling could contribute to the GATA6 deficiency and increased cell proliferation in PAH.

The BMP signaling via ALK1, BMPR2 and endoglin is mediated via canonical (SMAD1/5/9) and non-canonical pathways^40–42^. Interestingly, we found that silencing of SMAD1 with specific siRNA did not significantly reduce BMP10-dependent upregulation of GATA6 (Fig. 4L,M). Further analysis of non-canonical signaling pathways identified ERK1/2 as a regulator of BMP10-dependent GATA6 expression. Indeed, ERK1/2 suppression in HPAEC significantly reduced BMP10-dependent GATA6 protein and mRNA levels (Fig. 4N–P).

### GATA6 deficiency in PAEC and PASMC results in a reduced expression of BMP receptors

**Figure 5:**
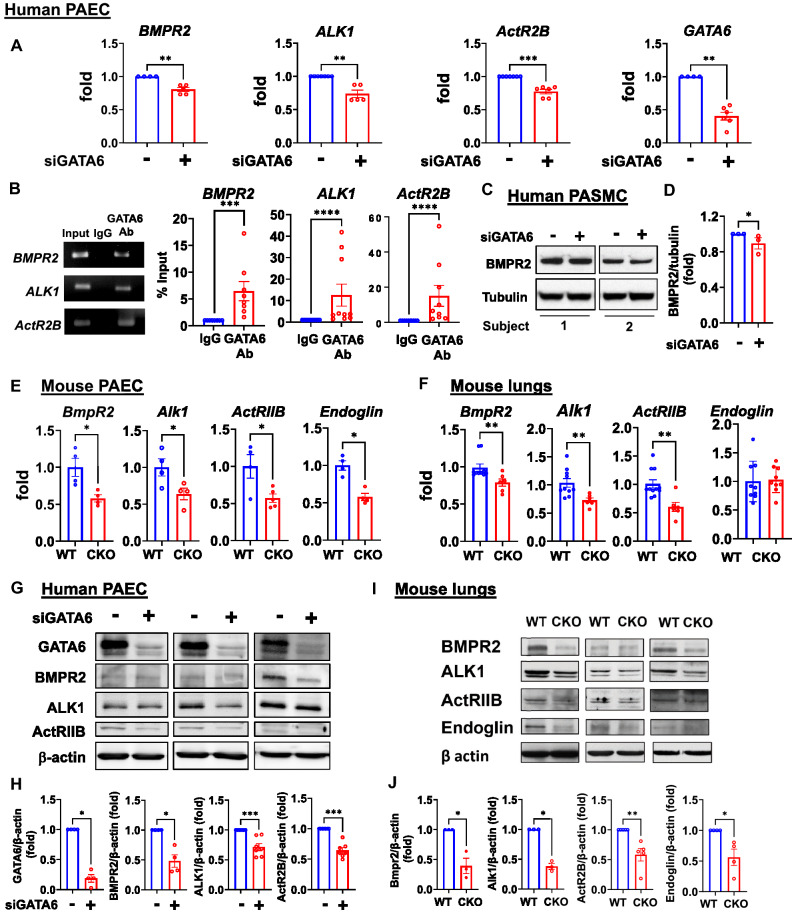
GATA6 deficiency in PAEC and PASMC results in loss of BMP receptors. (**A**) qPCR of HPAECs transfected with GATA6 or control scr siRNA ( − ) to measure indicated mRNA, each experiment was repeated at least three times. Data are means ± SE, n = 4–7. **p < 0.01, ***p < 0.001 by Mann Whitney U test. (**B**) Chromatin immune precipitation (ChiP) assay in HPAECs, n = 8–10. Representative gel images and data quantification are shown. Data are means ± SE, ***p < 0.001, ****p < 0.0001 by Mann Whitney U test. (**C,D**) Immunoblot analysis of control human PASMC transfected with siRNA GATA6 or control scr siRNA for 48 h. Data are means ± SE, 3 subjects/group, *p < 0.05 by Mann Whitney U test. Please see Fig. 2F,G for GATA6 immunoblots. (**E,F**) Expression of *BmpR2, Alk1*, *ActRIIB,* and *endoglin* measured by qPCR in PAEC (**F**) and whole lungs (**G**) from WT and Gata6 CKO mice. Data are means ± SE; E: n = 4–5/group; F: n = 6–11 mice/group. Male and female mice responded similarly. *p < 0.05, **p < 0.01 by Mann Whitney U test ((**E,F**) *BmpR2, Alk1*, and *ActRIIB*) and unpaired τ test (F *Endoglin*). (**G,H**) Control HPAECs transfected with siGATA6 or control scr siRNA ( − ) were assayed by immunoblot analysis to detect indicated BMP receptors. Values are means ± SE of the relative protein levels by densitometry, n = 4–7.*p < 0.05, ***p < 0.001 by Mann Whitney U test. (**I,J**) Immunoblot analysis of whole lung tissue from Gata6 CKO and WT mice. Values are means ± SE of the relative protein levels by densitometry, n = 3–5/group. Male and female mice responded similarly. *p < 0.05, **p < 0.01 by Mann Whitney U test. The original blots are presented in Supplementary Fig. S17.

We next investigated whether GATA6, in turn, modulates BMP signaling by testing the expression of the BMP receptors in GATA6-deficient HPAEC. Deficiency of GATA6 significantly reduced both mRNA and protein levels of *BMPR2, ALK1 and ActRIIB* in HPAEC (Fig. 5A,G,H). Chromatin immunoprecipitation analysis indicated that GATA6 acts as a direct transcriptional regulator for *BMPR2, ALK1,* and *ActR2B* in HPAEC (Fig. 5B). To determine whether canonical BMP10 pathway is involved in GATA6 regulation of the BMP receptors, we examined BMP10 stimulation of ID1/3 in GATA6-deficient HPAEC. As expected, BMP10 upregulated mRNA expression of ID1, while ID3 was not consistently induced. Deficiency of GATA6 did not affect the BMP10 response, suggesting that GATA6 does not interact with the canonical BMP10 pathway (Fig. S6). Depletion of GATA6 also moderately reduced BMPR2 levels in human PASMC (Fig. 5C,D). Likewise, expression of BMP receptors was reduced in PAEC and lung tissue extracts obtained from Gata6 CKO mice (Fig. 5E,F,I,J), suggesting that GATA6 positively regulates BMP signaling at the BMP receptors’ level. Consistent with our findings, it was recently reported that GATA6 transcriptionally regulates BMPR2 in PASMC^43^. These data further implied that GATA6 and BMP pathways cooperate to maintain the homeostasis of vascular cells.

### Endothelial GATA6 deficiency impairs GATA6 axis in PASMC

**Figure 6:**
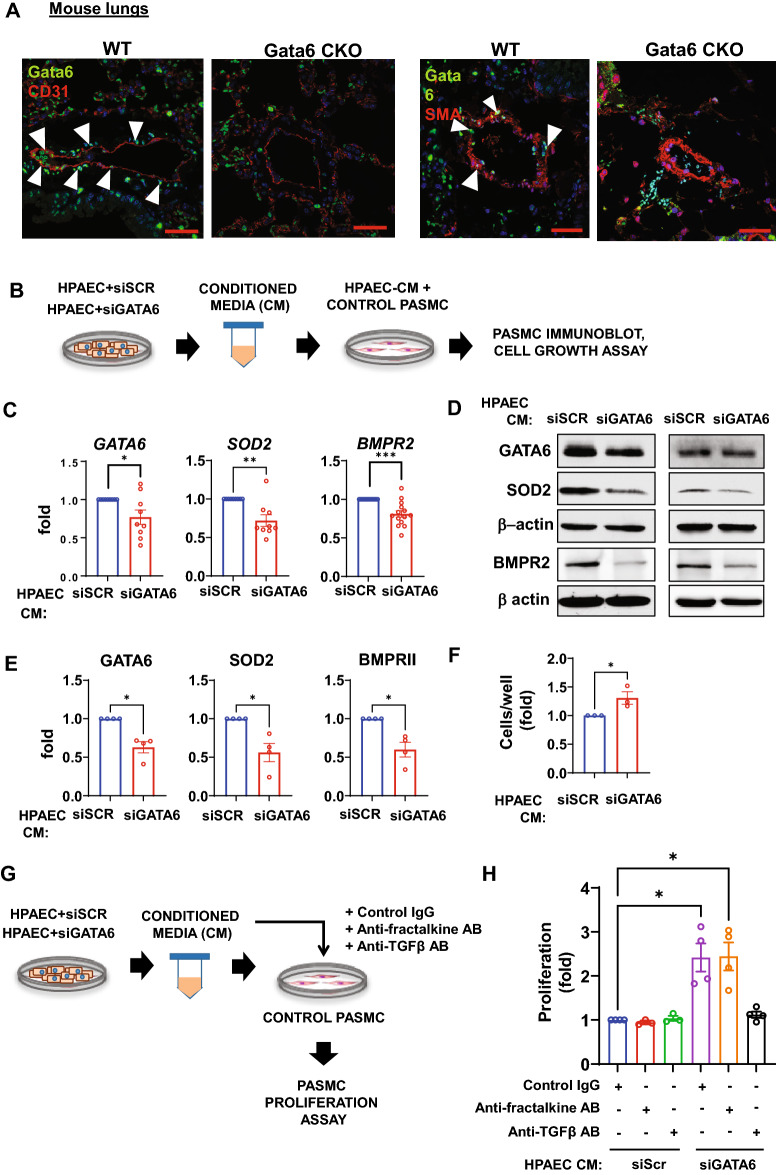
Endothelial GATA6 loss promotes GATA6 deficiency and increases growth of PASMC. (**A**) IHC stainings were performed to detect Gata6, CD31 and α-SMA in WT and Gata6 CKO mice; images are representative from 3 mice/group. Bar = 50 μm. White arrowheads indicate Gata6-positive cells. (**B**) Schematic representation of experiments (**C–F**). Media conditioned for 72 h by non-diseased human PAEC (HPAEC) transfected with GATA6 siRNAs or control scr siRNA was added to non-diseased distal human PASMC. (**C,D,E**) mRNA (**C**) and immunoblot analyses (**D,E**) of PASMC after 72 h incubation with HPAEC-conditioned media (CM) were performed. Data are means ± SE from n ≥ 3 repetitions. *p < 0.05, **p < 0.01, ***p < 0.001 by Mann Whitney U test. (**F**) Cell growth (cell counts) assay of PASMC after 48 h incubation with indicated HPAEC conditioned media (CM). Data are means ± SE, n = 3. *p < 0.05 by Mann Whitney U test. (**G,H**) PASMC were incubated for 48 h with indicated HPAEC conditioned media (CM) in the presence of 10 nM control IgG, anti-fractalkine antibody (AB), or anti-TGFβ AB. Schematic representation of experiment (**G**) and cell proliferation analysis (**H**) are shown. Data are means ± SE, n = 3–4/group. *p < 0.05 by Kruskal–Wallis test with post hoc Dunn’s test for multiple comparisons. The original blots are presented in Supplementary Fig. S18.

So far, our study has shown that GATA6 is downregulated in PAEC and PASMC and regulates the proliferative and oxidative status of these cells. Notably, mice with endothelial-specific Gata6 deficiency (Gata6 CKO) demonstrated reduced Gata6 expression in both endothelial and smooth muscle cells in small PAs (Fig. 6A). These observations led us to hypothesize that GATA6 deficiency is influenced by inter-cellular communications. To test whether PAEC-specific GATA6 deficiency is responsible for GATA6 downregulation in PASMC, we depleted GATA6 in HPAEC and treated non-diseased human PASMC with PAEC-conditioned media (PAEC-CM) (Fig. 6B). We found that the media from HPAEC transfected by siGATA6, but not control (scrambled) siRNA, decreased mRNA and protein levels of GATA6, SOD2, and BMPR2 in PASMC (Fig. 6C–E). Importantly, treatment with siGATA6 PAEC-CM induced growth of non-diseased PASMC (Fig. 6F). Together, these data strongly suggest that GATA6 controls PAEC-PASMC communication via paracrine factors, and endothelial GATA6 deficiency results in GATA6 loss and consequent proliferation of PASMC, exacerbating pulmonary vascular remodeling and PAH.

Based on published^44–46^ and our microarray data (Supplemental Table 1), we hypothesized that transforming growth factor β2 (TGFβ-2) and fractalkine (CX3CL1) could be paracrine factors driving PASMC proliferation induced by PAEC-specific GATA6 loss. Interestingly, treatment with anti-fractalkine neutralizing antibodies did not affect proliferation of PASMC in the presence of either siGATA6 PAEC-CM or siContr PAEC-CM (Fig. 6G,H, Fig S7). In contrast, anti-TGFβ neutralizing antibody prevented siGATA6 PAEC-CM-induced PASMC proliferation, while having no effect on the PASMC proliferation in the presence of siContr PAEC-CM (Fig. 6G,H, Fig S7). Further reinforcing our microarray analysis data showing TGFβ-2 among the top upregulated genes in GATA6-silenced HPAEC (Table S1), siRNA-induced GATA6 silencing in HPAEC resulted in induction of TGFβ-2, but not TGFβ-1 or TGFβ-3 mRNA (Fig. S8). Collectively, our data suggest that TGFβ-2 is upregulated in GATA6-silenced HPAEC and drives PASMC proliferation.

### Dimethyl fumarate (DMF) ameliorates pulmonary hypertension and reverses oxidative stress in Gata6 CKO mice

**Figure 7:**
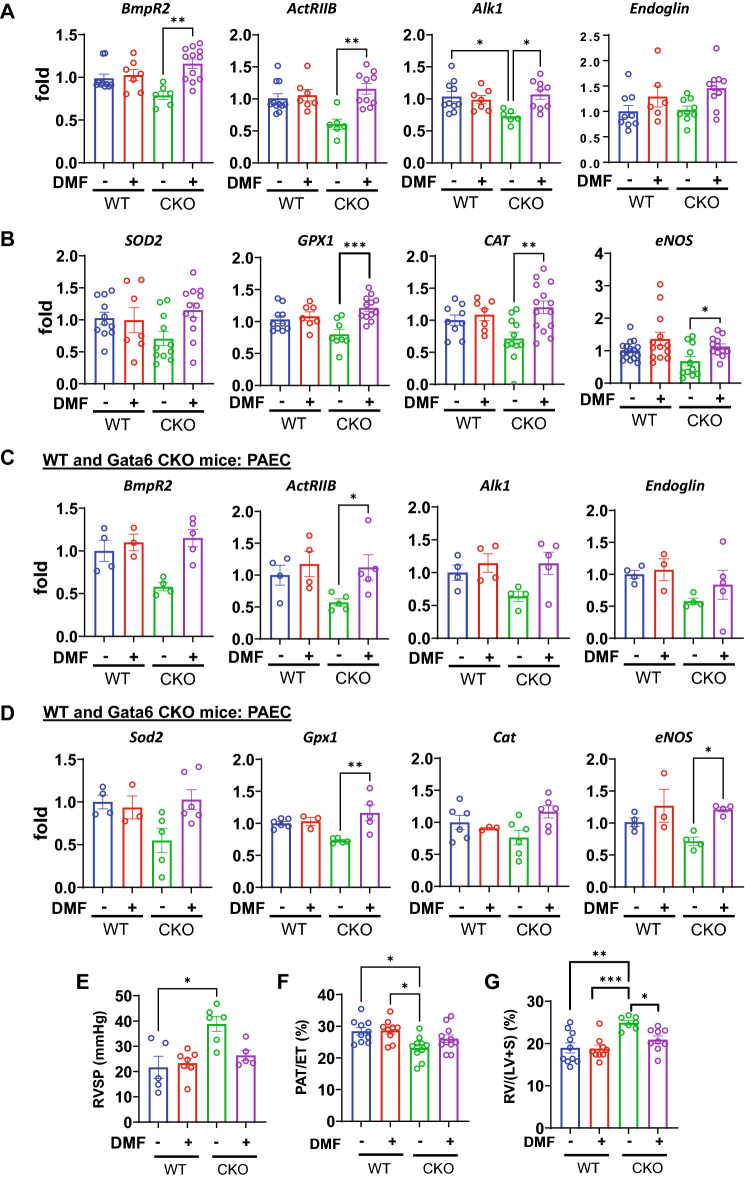
Treatment with DMF restores expression of the BMP receptors, reverses oxidative stress and pulmonary hypertension in Gata6 CKO mice. (DMF or vehicle were administered daily via i.p. injection for 3 weeks. (**A**) qPCR analysis of whole lung tissue from WT and Gata6 CKO mice treated with DMF or vehicle to detect expression of indicated BMP receptors. Data are means ± SE, n = 6–12, *p < 0.05. **p < 0.01 by Kruskal–Wallis test followed by Dunn’s multiple comparisons test (*BmpR2, ActRIIB, Alk1*) and one-way ANOVA followed by post hoc Tukey’s multiple comparison (*Endoglin*). (**B**) qPCR analysis of whole lung tissue from WT and Gata6 CKO mice treated with DMF or vehicle to detect expression of the antioxidant enzymes and *eNOS*. Data are means ± SE, n = 6–17, *p < 0.05., **p < 0.01, ***p < 0.001 by one-way ANOVA followed by post hoc Tukey’s multiple comparisons test (*SOD2, GPX1, CAT*) and Kruskal–Wallis test with post hoc Dunn’s multiple comparisons test (*eNOS*). (**C,D**) mRNA levels of indicated BMP receptors and antioxidant enzymes measured by qPCR in PAEC from WT and Gata6 CKO mice treated with DMF or vehicle. Data are means ± SE, n = 3–6 mice/group, *p < 0.05, **p < 0.01 by Kruskal–Wallis test with post hoc Dunn’s multiple comparisons test. (**E–G**) RVSP, pulmonary acceleration time as a fraction of ejection time (PAT/ET) and Fulton index (RV/[LV + S]) were evaluated in WT and Gata6 CKO mice in the presence or absence of DMF. Data are means ± SE. n = 5–11 mice/group. *p < 0.05, **p < 0.01. ***p < 0.001 by Kruskal–Wallis test with post hoc Dunn’s multiple comparisons test (RVSP) and one-way ANOVA followed by post hoc Tukey’s multiple comparisons test (PAT/ET and RV/(LV + S).

The complexity of PAH, including impaired redox homeostasis, abnormal vascular remodeling, and immune cell activation suggests that a therapeutic agent capable of modulating several key pathways would be an attractive addition to the treatment regimen of PAH. The transcriptional regulator nuclear factor (erythroid-derived 2)-related factor 2 (NRF2) is a key regulator of the antioxidant genes^47^. Importantly, NRF2 function is linked to NFκB signaling with activation leading to an anti-inflammatory response. In support of NRF2-based therapy in PAH, bardoxolone methyl (CDDO-Me), a NRF2 inducer has shown encouraging results in a Phase II study in PAH (https://pulmonaryhypertensionnews.com/2015/10/27/reatas-bardoxolone-methyl-pah-therapy-shows-promise-initial-phase-2-data/). DMF (BG-12, Tecfidera^®^), another inducer of NRF2, has recently been approved in the US for the treatment of multiple sclerosis^48^. DMF has an excellent safety record in MS and two decades of use in psoriasis, also an inflammatory condition. DMF has been evaluated in experimental models of PH^49^. DMF mitigated PH by targeting multiple signaling pathways including oxidative damage, inflammation, and fibrosis. To test the therapeutic effects of DMF on pulmonary hypertension in Gata6 CKO mice, we first assessed the effect of DMF on oxidative stress. DMF or a vehicle control were administered daily via i.p. injection for 3 weeks, as previously described^49^. DMF restored the mRNA expression of the *BMP* receptors (Fig. 7A) as well as expression of antioxidant enzymes (Fig. 7B). DMF also restored eNOS expression levels, previously shown to be downregulated in Gata6 CKO mice^15^. The beneficial effects of DMF on the expression of the *BMP* receptors, antioxidant enzymes and *eNOS* were further confirmed in PAECs isolated from the DMF treated Gata6 CKO mice (Fig. 7C,D). To test whether DMF directly affects expression of the BMP pathway receptors, GATA6-deficient HPAEC were treated with DMF. DMF modestly increased expression of *ALK1, ACVR2B, BMPR2*, and *endoglin* in Scr siRNA treated HPAEC, and reversed the downregulation of the receptors in GATA6 siRNA treated cells, without affecting GATA6 expression (not shown) (Fig. S9). Furthermore, DMF restored the oxygen consumption rate in GATA6-deficient PAEC (supplemental Fig. S10). These data suggest that DMF acts via a GATA6-independent mechanism to upregulate BMP receptor expression and reverse oxidative stress and mitochondrial dysfunction. Furthermore, DMF decreased proliferation and induced apoptosis of human PAH PASMC (Fig. S11).

To test the therapeutic effects of DMF on pulmonary hypertension in Gata6 CKO mice, we measured right ventricle systolic pressure, as well as pulmonary acceleration time as a fraction of ejection time (PAT/ET) and the weight ratios of the right ventricle to the left ventricle plus septum. DMF normalized pulmonary pressure and partially reversed right heart hypertrophy in Gata6 CKO mice (Fig. 7E–G, Fig. S12). Together, these data demonstrate that treatment with DMF resolves oxidative stress, restores BMP receptors, and reverses PH caused by endothelial Gata6 loss.

## Discussion

**Figure 8:**
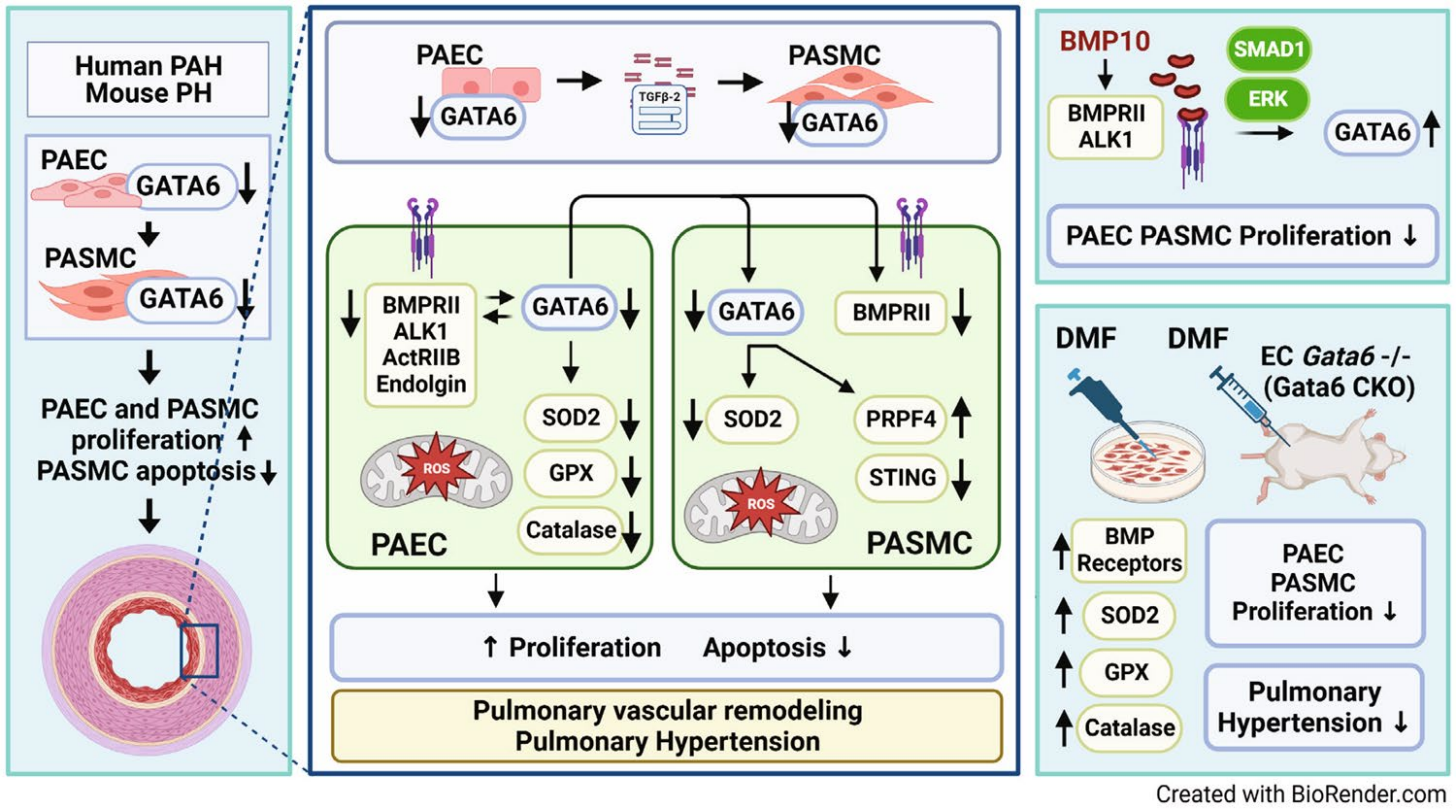
Graphical representation of the role of GATA6 in coordinating cross-talk between BMP10 and oxidative stress axis in PAH. GATA6 is an activator of anti-oxidant enzymes and its deficiency in PAEC and PASMC induces oxidative stress and mitochondrial dysfunction. BMP10 induces expression of GATA6 through the ALK1, BMPRII, ENG and ERK pathway. GATA6, in turn, transcriptionally activates BMP receptors in PAEC. Endothelial GATA6 regulates PASMC function via paracrine factors. TGFβ2 secreted by GATA6 deficient PAEC induces PASMC proliferation. Administration of dimethyl fumarate (DMF) to mice with endothelial Gata6 loss restores expression of BMP receptors, resolves oxidative stress, and reverses PH.

Here we report novel mechanism(s) of GATA6 regulation and function in pulmonary vasculature and provide the evidence demonstrating the attractiveness of the GATA6 axis as a molecular target pathway for therapeutic intervention in PAH. Our novel findings include that: (i) GATA6 acts as a transcription factor and direct positive regulator of anti-oxidant enzymes, and its deficiency in PAH/PH pulmonary vascular cells induces oxidative stress and mitochondrial dysfunction; (ii) GATA6 forms bi-directional cross-talk with the BMP10/BMP receptors axis, and its loss in PAH/PH pulmonary vascular cells supports BMPR deficiency; (iii) GATA6 plays an important role in pulmonary vascular cell communication, and endothelial GATA6 deficiency results in GATA6 loss in PASMC; and (iv) targeting the Gata6 axis with DMF resolves oxidative stress and BMPR deficiency and ameliorates experimental PH in mice (Fig. 8).

The current paradigm of PAH is that early injury of PAEC leads to positive selection of hyper-proliferative PAEC and reprogramming of PASMC to a proliferative/apoptosis resistant phenotype, consequent remodeling of small pulmonary arteries, and pulmonary hypertension^7^. Oxidative stress is recognized as a major driver of the pro-proliferative and anti-apoptotic changes in PAH PAEC and PASMC^50–52^. Here we show that GATA6 is a key transcription activator for the two central mitochondrial enzymes, SOD2 and GPX-1, required for the anti-oxidant response in PAEC. Loss of GATA6 in PAH PAEC caused excessive cellular and mitochondrial ROS production due to the reduced activity of SOD2, GPX1, as well as catalase. *SOD2* is a putative tumor-suppressor gene, deficiency of which is linked to proliferative diseases including PAH and cancers. In addition to oxidative stress^53,52^, decreased expression of SOD2 promotes PH by activating HIF1α, causing a metabolic shift to glycolysis, increased proliferation, and reduced apoptosis^54,55^. Suggesting a mechanistic link between GATA6 and SOD2, we have previously reported increased Hif1α, Hif1β, and Hif2α expression in the lungs of Gata6CKO mice^15^. Indeed, our new data show that GATA6 deficiency results in loss of SOD2 in human and mouse pulmonary vascular cells, and that restoration of GATA6 in PAH PAEC and PASMC reduces proliferation of both cell types. Furthermore, re-expression of GATA6 in PAH PAEC correlated with increased SOD2 levels, suggesting that SOD2 could contribute to the anti-proliferative effects of GATA6 in these cells. Intriguingly, restoration of GATA6 in PAH PASMC, while suppressing proliferation and inducing apoptosis, was unable to restore SOD2 expression, suggestive that there are PASMC-specific downstream mediators of the GATA6 anti-proliferative function in PAH.

Further analyses identified two potential novel mediators of the GATA6-dependent hyperproliferative and anti-apoptotic phenotype in PAH PASMC: STING (also known as TMEM173) and PRPF4. While it’s never been studied in PAH pulmonary vasculature, the stimulator of *IFN* genes STING is well-known for its role in innate immunity regulation as an inducer of type I interferon production in response to intracellular pathogens^56,57^. Importantly, a recent study in cancer cells has uncovered an additional novel role of STING as a regulator of cell cycle and a potential tumor suppressor gene^58^. Interestingly, we found that STING expression was reduced in PAH PASMC, suggesting that the pro-proliferative effects induced by GATA6 deficiency might be, in part, mediated by the insufficiency of STING. Another identified target of GATA6 with a potential role in the hyperproliferative phenotype of PAH PASMC was the pre-mRNA processing factor 4, PRPF4. PRPF4 is involved in spliceosome assembly by regulating pre-mRNA splicing as component of the U4/U6-U5 tri-snRNP complex^59–61^. PRPF4 has been associated with increased cell growth and reduced apoptosis in cancer cells, however the specific role of PRPF4 in those processes remains unclear^62,63^. PRPF4 expression was increased in PAH PASMC, suggesting that PRPF4 may also contribute to the disease-specific hyperproliferative phenotype. Given the potentially important role of STING and PFPR4 in mediating cell proliferation and apoptosis, further work on their involvement in PAH is warranted.

BMP9 and − 10 are present in the circulation, play a central role in regulating endothelial cell homeostasis^64^, and induce quiescence of endothelial cells^26,38^, but the underpinning mechanism has not been fully elucidated. The mechanistic importance of BMP9 deficiency in portopulmonary hypertension had been recently reported by Paul Yu’s laboratory^35^, and loss-of-function mutations in BMP9 and GDF2 are identified in PAH and result in reduced BMP9 and BMP10 circulating levels in PAH patients^29,65^. Paradoxically, selective Bmp9 inhibition in rodents appeared to be partially protective against experimental PH^66^ indicative of the complexity of BMP9 signaling. Our data have revealed a novel positive feedback loop between the BMP10 and the GATA6 axis. We now report that in both, human and mouse PAECs, BMP10 induces expression of GATA6, and that ALK1, BMPR2 and Endoglin are necessary for this induction. Further, we show that GATA6 transcriptionally activates *ALK1, BMPR2,* and *ActRII* genes and that GATA6 deficiency leads to the decreased mRNA and protein levels of these BMP receptors. Together, our data suggest functional codependence of the GATA6 and the BMP10 pathways in mediating PAEC and PASMC homeostasis in a non-diseased state and protection against hyper-proliferation and impaired apoptosis in PAH.

Cross-talk between endothelial and smooth muscle cells in pulmonary vasculature has long been recognized as a critical event during pathological vessel remodeling in PAH^67^. Although progress has been made in identifying factors produced by PAEC or PASMC and facilitating such a communication^68–71^, more work is needed to fully elucidate this process. Consistent with the previous reports indicating the increased ability of PAH PAEC to secrete mitogens for PASMC^70^, here we showed that GATA6-deficient PAEC not only increased proliferation of PASMC, but also induced other pathological changes, including downregulation of GATA6, SOD2, and BMPR2 in PASMC. The GATA6 dependent secreted factors, however, remain to be characterized.

Taken together, our work has demonstrated wide-ranging detrimental effects of GATA6 deficiency on the function of both PAEC and PASMC in the context of PAH. These effects may be specific to pulmonary vasculature, as distinct consequences of endothelial GATA6 deletion were reported in carotid artery ligation or femoral artery wire-injury models^72^. In those models knock-out of endothelial Gata6 elicited a protective effect by reducing neointima formation in both experimental models. The authors linked reduced levels of PDGF-B to the anti-proliferative effects associated with endothelial Gata6 loss. In our study, we were not able to reproduce those findings in GATA6-deficient human PAEC (data not shown), and on the contrary, we observed a moderate increase of *PDGF-B* mRNA in response to the reduced levels of GATA6. The reason for this discrepancy is unclear; however, it is possible that endothelial cells from different vascular beds differ with respect to the effects of GATA6 loss.

Our study, however, have limitations. Specifically, SSc-PAH PAEC and PASMC were not available in the required quantities to perform all experiments. The SSc-PAH is a sub-group of rare disease PAH that occurs in patients with rare disease SSc. In 2013, the prevalence of SSc in United States was ~ 240 cases per million; and the prevalence of PAH in patients with SSc is 8–14%^73^. This makes patient-derived samples very rare and limits our access to the patients-derived resident PA cells of early passage available for research. However, we strongly believe that including the cells that were available to us into select experiments together with cells from other PAH patients improves our understanding about the pathobiology of SSc-PAH and overall PAH.

Published studies and our new data strongly suggest that GATA6 acts as a multi-cellular signaling hub regulating pulmonary vascular homeostasis, and that dual PAEC-PASMC GATA6 deficiency promotes pulmonary vascular remodeling and PH via disturbing the balance among major signaling pathways regulating cell proliferation and survival. Thus, targeting GATA6 deficiency may provide a major therapeutic advance for patients with PAH. Here we report that DMF treatment was very effective in reversing hemodynamic changes, reducing oxidative damage, and restoring expression of the Bmp receptors in Gata6 CKO mice. These findings are consistent with the previously shown beneficial therapeutic effects of DMF in hypoxia and SU5416/hypoxia mouse models of PH^49^. While NRF2-inducing properties of DMF or CDDO-Me are frequently emphasized, it is important to recognize that these compounds have multiple, not yet fully understood, modes of action, which may account for their beneficial effects in multifactorial diseases such as PAH. Given the cytoprotective and immunomodulatory effects of DMF and its proven safety record in other chronic diseases^74,75^, DMF may be an attractive therapeutic strategy for PAH.

### Study approval

The use of unidentified human tissue and cells in this study is considered “no human subjects research” by IRB. Institutional approval was granted by protocol #458 of the University of Pittsburgh CORID. All animal experimental procedures were performed in accordance to protocol # TR201900057 reviewed and approved by the Boston University Institutional Animal Care and Use Committee. The investigation conformed to the Guide for the Care and Use of Laboratory Animals, published by the US National Institutes of Health (NIH).

## Supplementary Information

Supplementary Figures.

Supplementary Figures.

Supplementary Tables.

Supplementary Tables.

## Data Availability

All data generated or analyzed during this study are included in this published article and its supplementary information files.
