# Supplementary material for: GATA6 coordinates cross-talk between BMP10 and oxidative stress axis in pulmonary arterial hypertension
Source: Sci Rep. 2023 Apr 22;13:6593. doi: 10.1038/s41598-023-33779-8 (PMC10122657; doi:10.1038/s41598-023-33779-8)
Supplement: Supplementary file 1 — Supplementary Figures. [file 41598_2023_33779_MOESM1_ESM.pdf]

SUPPLEMENTAL FIGURES

GATA6 coordinates cross-talk between BMP10 and oxidative stress axis in pulmonary arterial hypertension

Tetsuo Toyama, Tatiana V. Kudryashova, Asako Ichihara, Stefania Lenna, Agnieszka Looney, Yuanjun Shen, Lifeng Jiang, Leyla Theos, Theodore Avolio, Derek Lin, Ulas Kaplan, Grace Marden, Vrinda Dambal, Dmitry Goncharov, Horace Delisser, Robert Lafyatis, Francesca Seta, Elena A. Goncharova, Maria Trojanowska

Supplemental Figures:

Figure S1, related to Figure 1.
Gene expression of the antioxidant enzymes measured by qPCR in PAECs isolated from GATA6 KO mice

Figure S2.
GATA6 loss induces oxidative stress and mitochondrial dysfunction in human and mouse PAEC and PASMC.

Figure S3, related to Figure 4.
GATA6 loss increases mitochondrial membrane potential, reduces respiration in human PAEC.

Figure S4, related to Figure 3.
Restoration of GATA6 induces PAH PASMC apoptosis.

Figure S5, related to Figure 4.
BMP9 induces GATA-6 expression in human PAEC.

Figure S6, related to Figure 5.
GATA6 depletion does not affect BMP10 stimulation of ID1/ID3.

Figure S7, related to Figure 6.
PAEC-secreted TGFβ promotes proliferation of human PASMCs

Figure S8, related to Figure 6. GATA6-dependent expression of TGFβ isoforms in HPAECs

Figure S9, related to Figure 7.
DMF acts downstream of GATA6 to restore BMP receptors

Figure S10, related to Figure 7
DMF restores oxygen consumption rate in HPAEC transfected with siGATA6 siRNA

Figure S11, related to Figure 7
DMF inhibits growth and induces apoptosis in human PAH PASMC

Figure S12, related to Figure 7.
Echocardiography analysis of WT and CKO mice treated with diluent or DMF

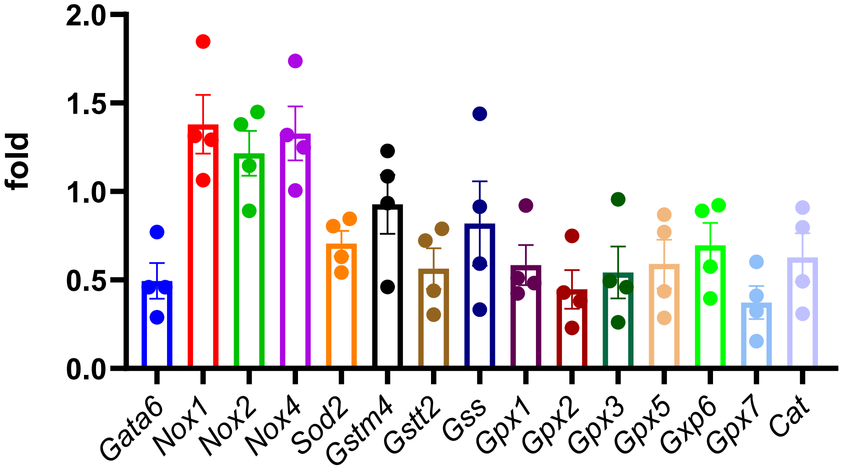

Figure S1, related to Figure 1.
Gene expression of the antioxidant enzymes measured by qPCR in PAECs isolated from GATA6 KO mice

mRNA levels were measured by qRT-PCR, and the value for each individual mouse PAEC was normalized to the mean level in WT mouse PAEC taken as 1 fold. * p<0.05; ** p<0.01 vs. control PAECs. 0.01 by Kruskal-Wallis test with post-hoc Dunn's correction for multiple comparisons.

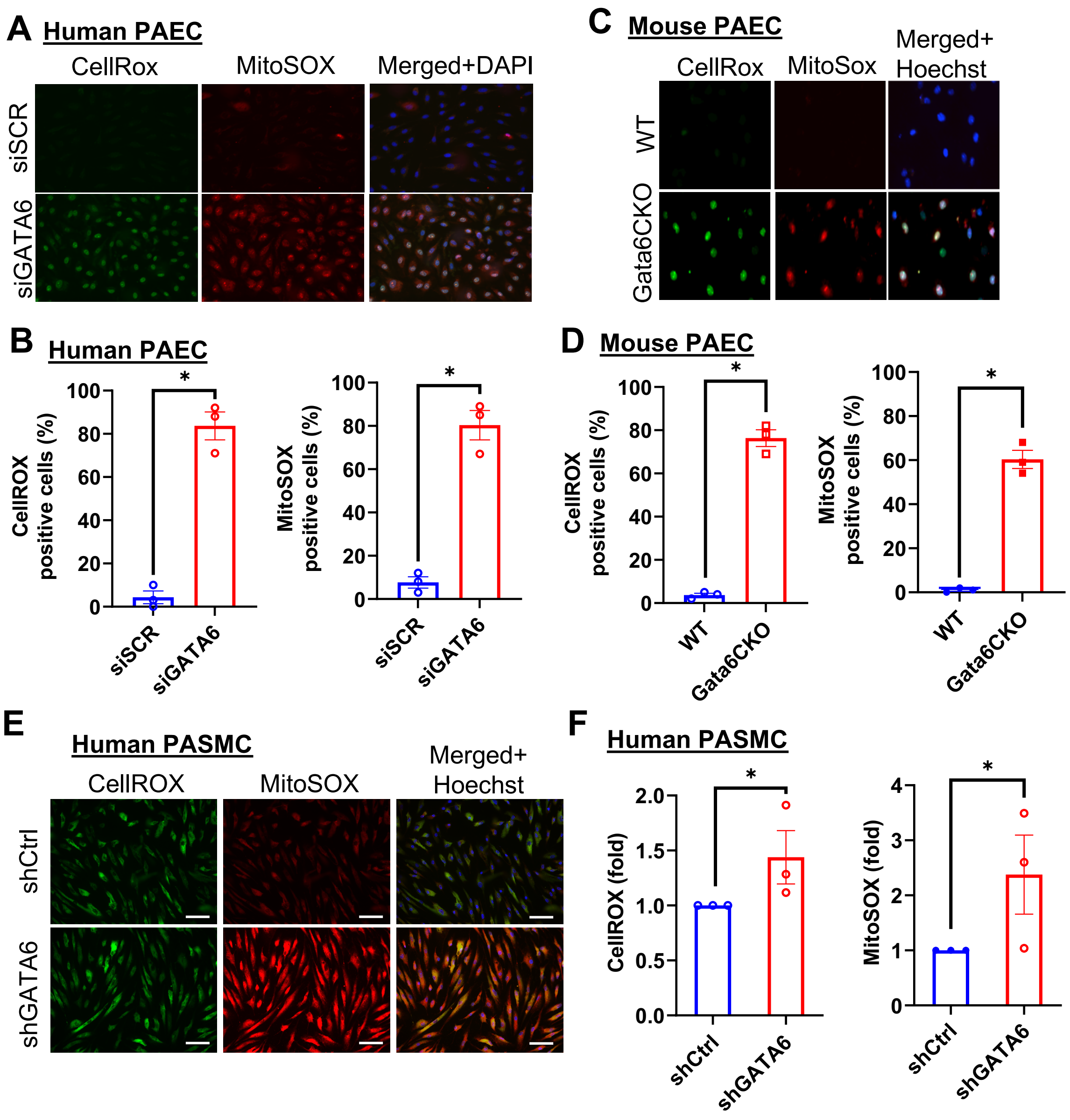

Figure S2.
GATA6 loss induces oxidative stress and mitochondrial dysfunction in human and mouse PAEC and PASMC.

A-D. Cellular ROS levels in HPAECs transfected with siGATA6 or scr siRNAs and in Gata6 CKO mice or WT measured with the mitochondrial superoxide (MitoSOX) probe (red) and general oxidative stress (CellROX) probe (green). Quantification of positive cells measured from 5 fields of view per group. *p<0.05 by Mann-Whitney U test.
E-F. Cellular ROS levels were measured in human non-diseased PASMC infected with lentivirus producing shGATA6 or scr (shCtrl) shRNAs with the MitoSOX probe (red) and CellROX probe (green). Nuclei were stained with Hoechst (blue). Images are representative from three separate experiments, each performed on the cells from different human subject. CellROX and MitoSOX signal intensity was measured and quantified from 3 subjects/group, 3 fields of view/subject, minimum of 27 cells per field. Bar equals 100 μm. Data are means±SE, fold to shCtrl group (-), *p<0.05 by Mann-Whitney U test.

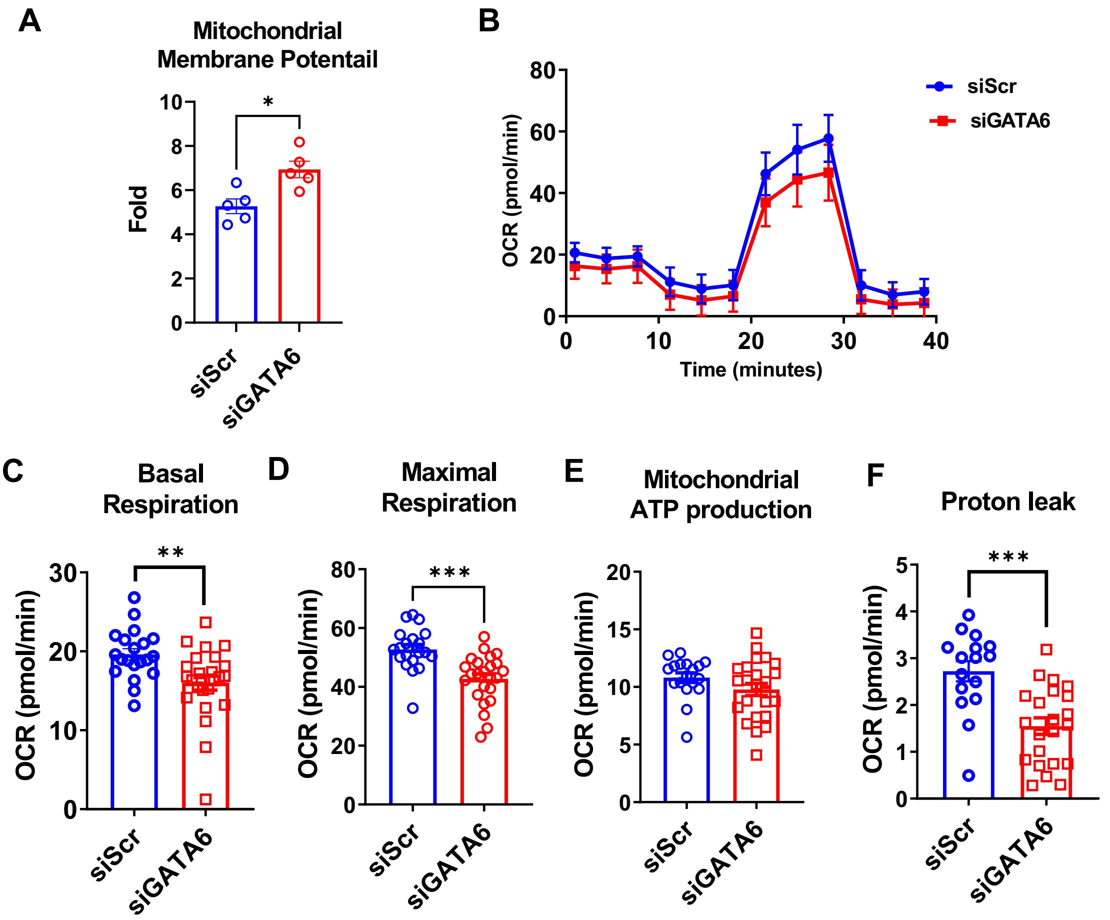

Figure S3, related to Figure 4.
GATA6 loss increases mitochondrial membrane potential, reduces respiration in human PAEC

A. Mitochondrial membrane potential (MMP) in HPAEC transfected with siGATA6 or scr siRNAs. Data are means±SD; n=5/group; *p<0.05 by Mann Whitney U test.
B-D. Oxygen consumption rate (B), basal (**C**) and maximal (**D**) respiration following FCC treatment, mitochondrial ATP production (**E**), and proton leak (**F**) of HPAEC transfected with siGATA6 or control scr siRNAs (siScr). The experiments were repeated at least 3 times. Data shown as mean ± SE, n=12-24 *p<0.01, **p<0.01, ***p<0.001 by unpaired τ test.

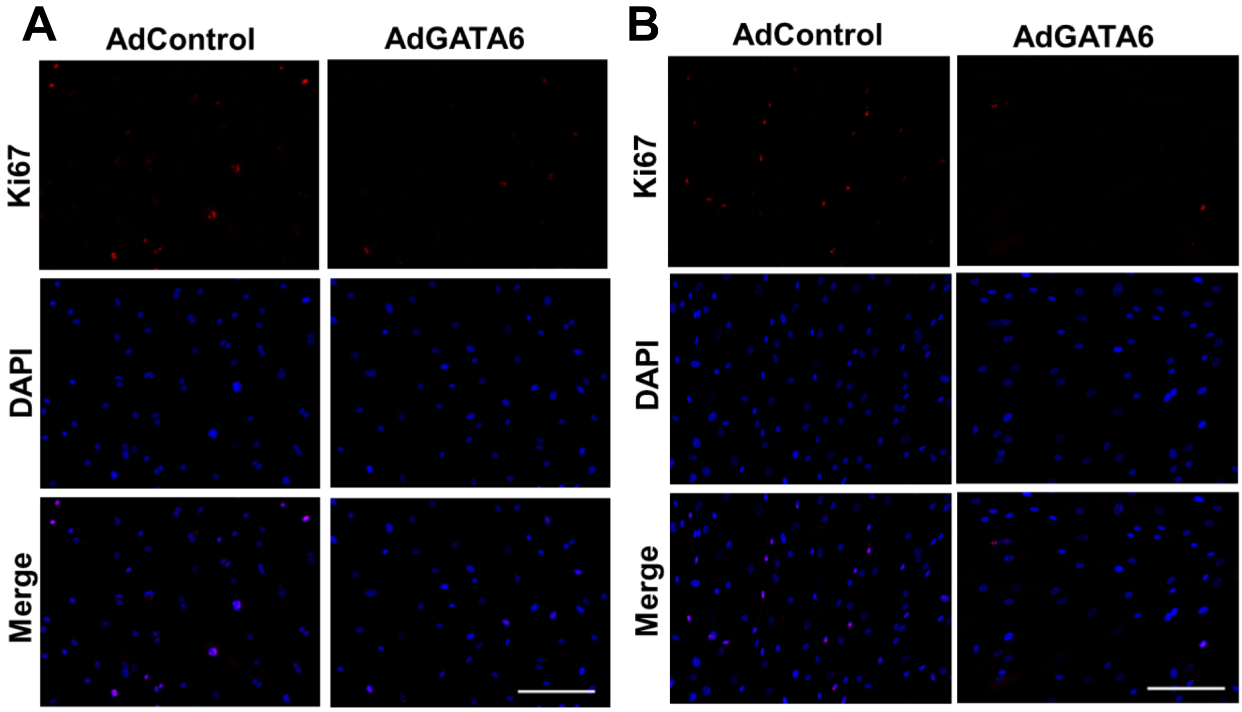

Figure S4. Restoration of GATA6 induces PAH PASMC apoptosis

Representative images of human PAH PAECs (A) and human PAH PASMCs (B) transfected with 10 MOI of control adenovirus (AdControl) or AdGATA6 for 48 hours followed by proliferation (Ki67) assay. Red - Ki67; blue - DAPI. Bar equals 200 µm.

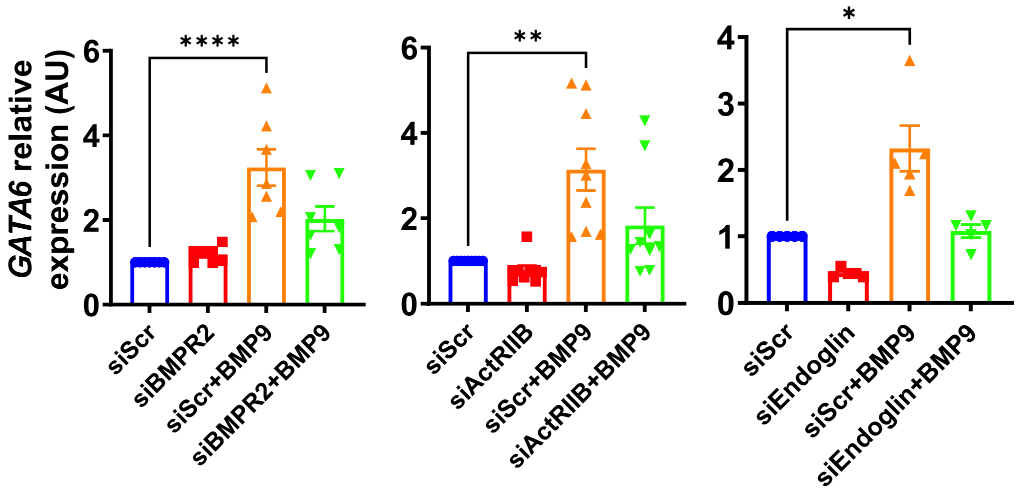

Figure S5, related to Figure 4.
BMP9 induces GATA-6 expression in HPAEC

HPAECs were transfected with control (scrambled), BMPR2, ActRIIB, or endoglin siRNA, separately or in combination, and then treated with BMP9 for 6h. *GATA6* mRNA level was measured by qPCR. Data shown as means±SE, n=7-9/group. Each experiment was repeated at least three times. *p<0.05, **p<0.01, ****p<0.0001 by Kruskal-Wallis test with post-hoc Dunn's correction for multiple comparisons.

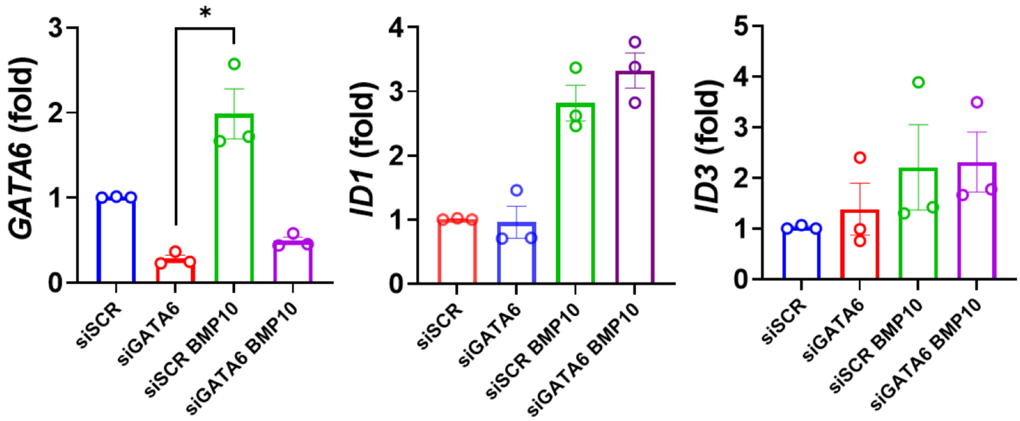

Figure S6, related to Figure 5.
GATA6 depletion does not affect BMP10 stimulation of ID1/ID3.

HPAEC were transfected with SCR siRNA (siSCR) or GATA6 siRNA (siGATA6). 24 hours after the transfection, cells were serum starved with 0.1% BSA overnight, then, treated with 10ng of BMP10 for 24 hours in the presence of 0.1% BSA. *GATA6*, *ID1*, and *ID3* mRNA levels were measured with qPCR. The experiment was repeated at least three times and the data is shown as means±SE. *p<0.05 by Kruskal-Wallis test followed by post hoc Dunn's multiple comparisons test.

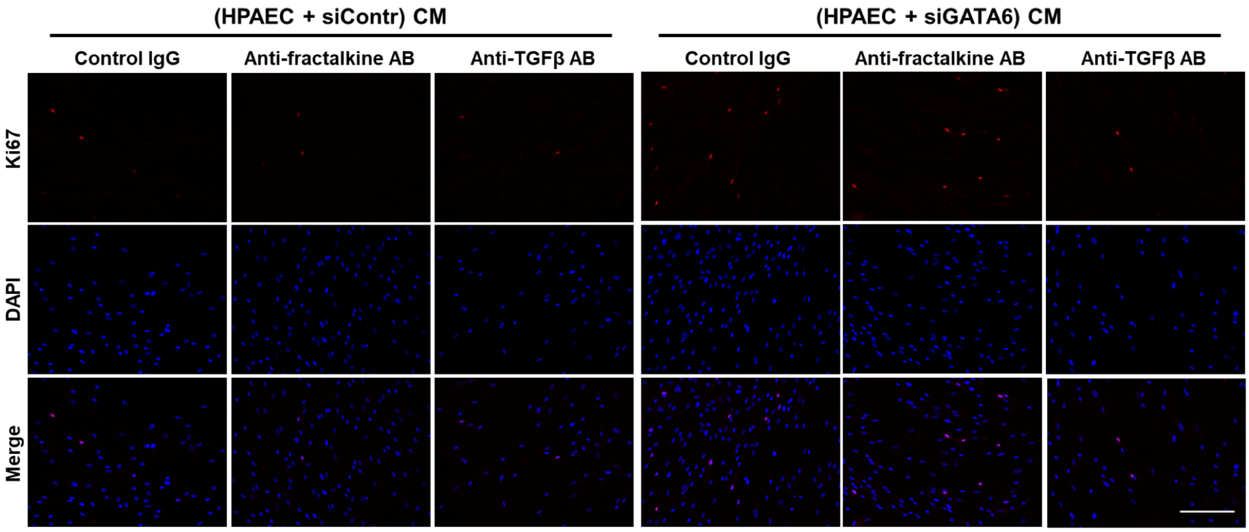

Figure S7, related to Figure 6.
PAEC-secreted TGFβ promotes proliferation of human PASMCs

Ki67 (red) and DAPI staining (blue) performed on PASMC incubated for 48h with indicated HPAEC conditioned media (CM) in the presence of 10 nM control IgG, anti-fractalkine antibody (AB), or anti-TGFβ AB. Images are representative of minimum of 3 independent experiments. Bar equals 200 µm.

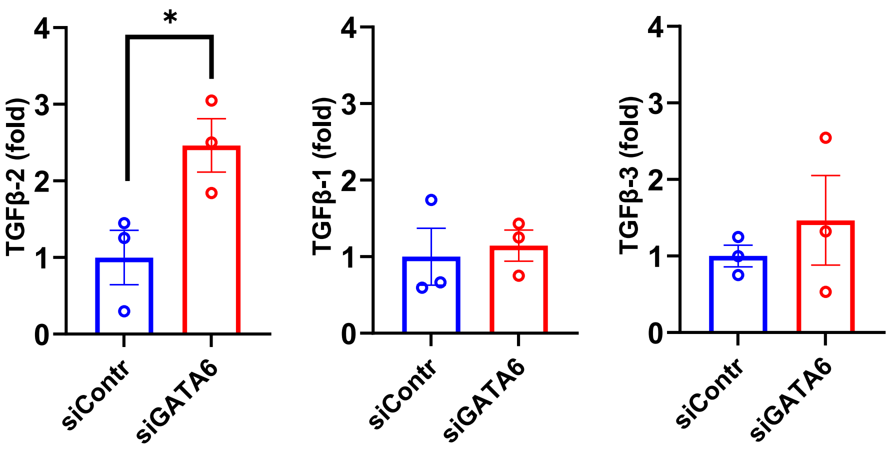

Figure S8, related to Figure 6.
GATA6-dependent expression of TGFβ isoforms in HPAECs

HPAECs were transfected with siRNA GATA6 (siGATA6) or control scrambled siRNA (siContr). 48 hours later, qPCR analysis was performed to detect indicated mRNA levels. Data are means±SE; n=3/group, *p<0.05 by Mann-Whitney U test.

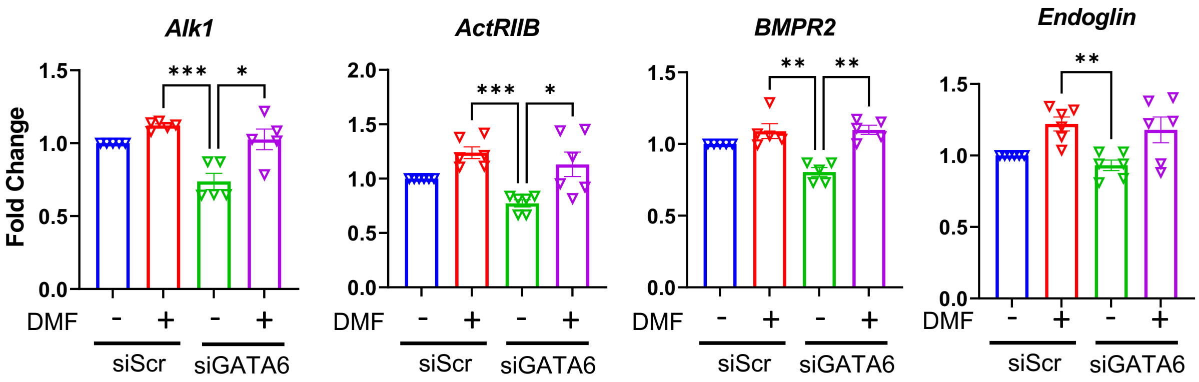

Figure S9 related to Figure 7.
DMF acts downstream of GATA6 to restore BMP receptors

HPAECs were transfected with GATA6 siRNA in the presence or absence of DMF. Gene expression were measured by qPCR. Data shown as means±SE. n=5-6/group. *p<0.05, **p<0.01, ***p<0.001 by Kruskal-Wallis test with Dunn's post-hoc correction for multiple comparisons.

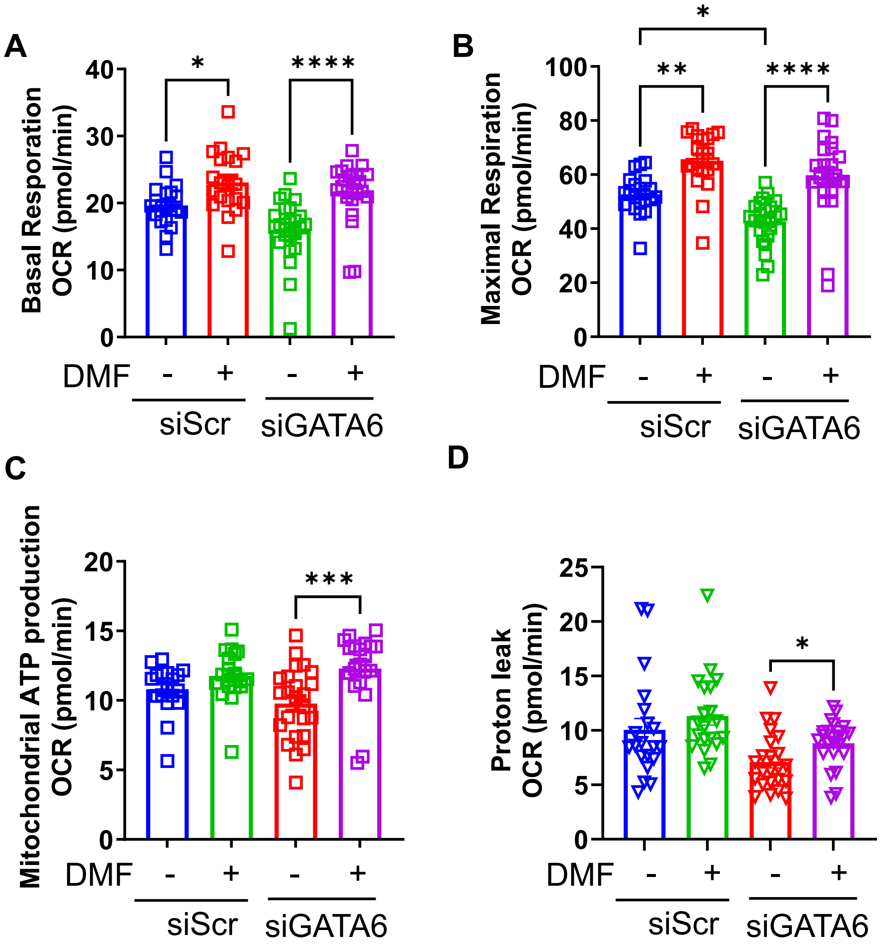

Figure S10, related to Figure 7.
DMF restores oxygen consumption rate in HPAEC transfected with siGATA6 siRNA.

A. Basal and **B.** maximal respiration following FCC treatment. **C.** Mitochondrial ATP production. **D.** Proton leak. Data are means±SE, n=20-25/group, *p<0.05, **p<0.01, ***p<0.001, ****p<0.0001 by Kruskal-Wallis test with Dunn's post-hoc correction for multiple comparisons.

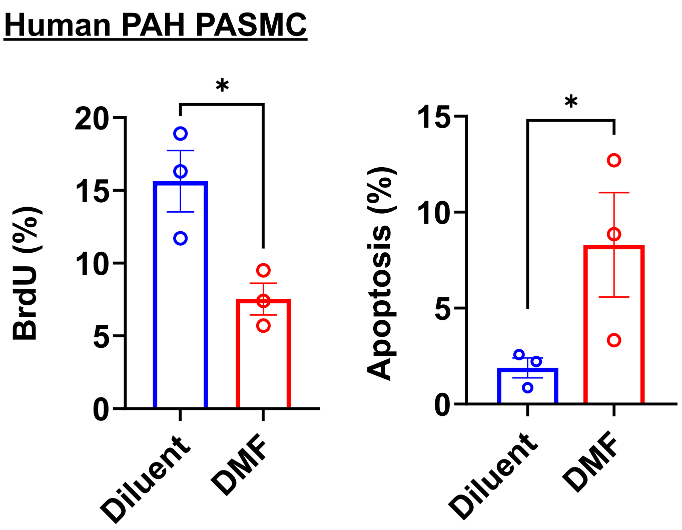

Figure S11, related to Figure 7.
DMF inhibits growth and induces apoptosis in human PAH PASMC

Cells were treated with 5μM DMF or diluent for 24h, and then analysis of cell proliferation (DNA synthesis, BrdU) (left) and apoptosis (In *Situ* Cell death detection kit (Sigma) according to the manufacturer's protocol) (right) were performed. Data are means±SE; n=3 subjects/group; *p<0.05 by Mann-Whitney U test.

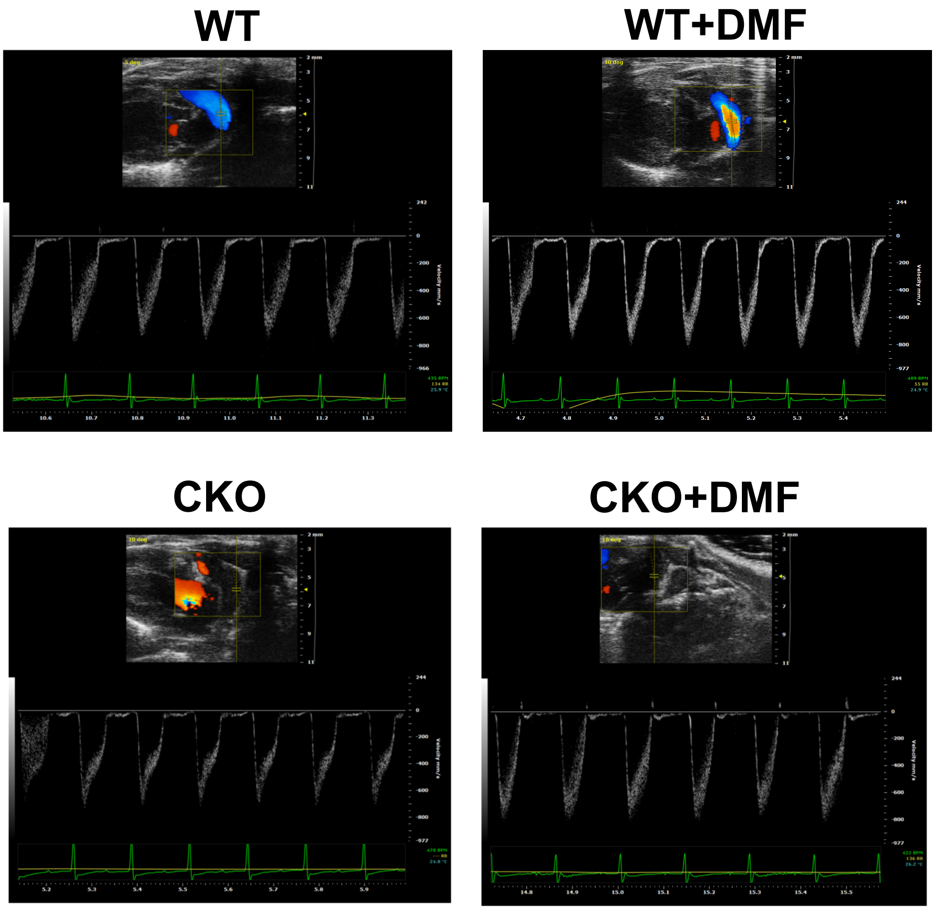

Figure S12, related to Figure 7.
Echocardiography analysis of WT and CKO mice treated with diluent or DMF

Echocardiography analysis of WT and CKO mice treated with DMF or diluent performed using the Vevo 770 High-Resolution Imaging System. Images are representative from 5-11 mice/group.
