# Supplementary material for: GATA6 coordinates cross-talk between BMP10 and oxidative stress axis in pulmonary arterial hypertension
Source: Sci Rep. 2023 Apr 22;13:6593. doi: 10.1038/s41598-023-33779-8 (PMC10122657; doi:10.1038/s41598-023-33779-8)

Figure S13a. Original images for Figure 1B.

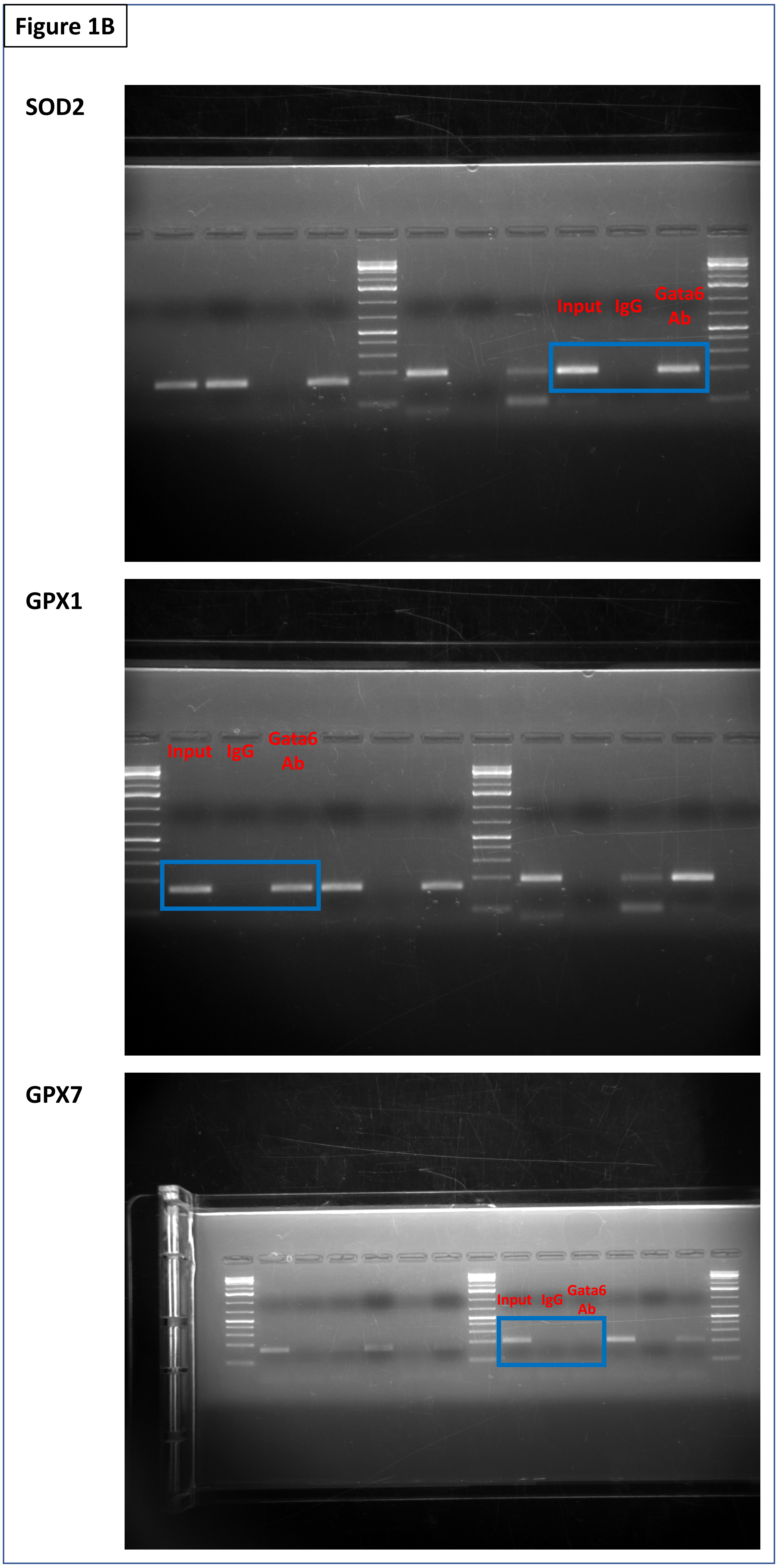

Figure S13b. Original immunoblot images for Figure 1C and 1E.

The full length immunoblots for Figures 1C, and E could not be provided because blots were cut prior to hybridization with antibodies.

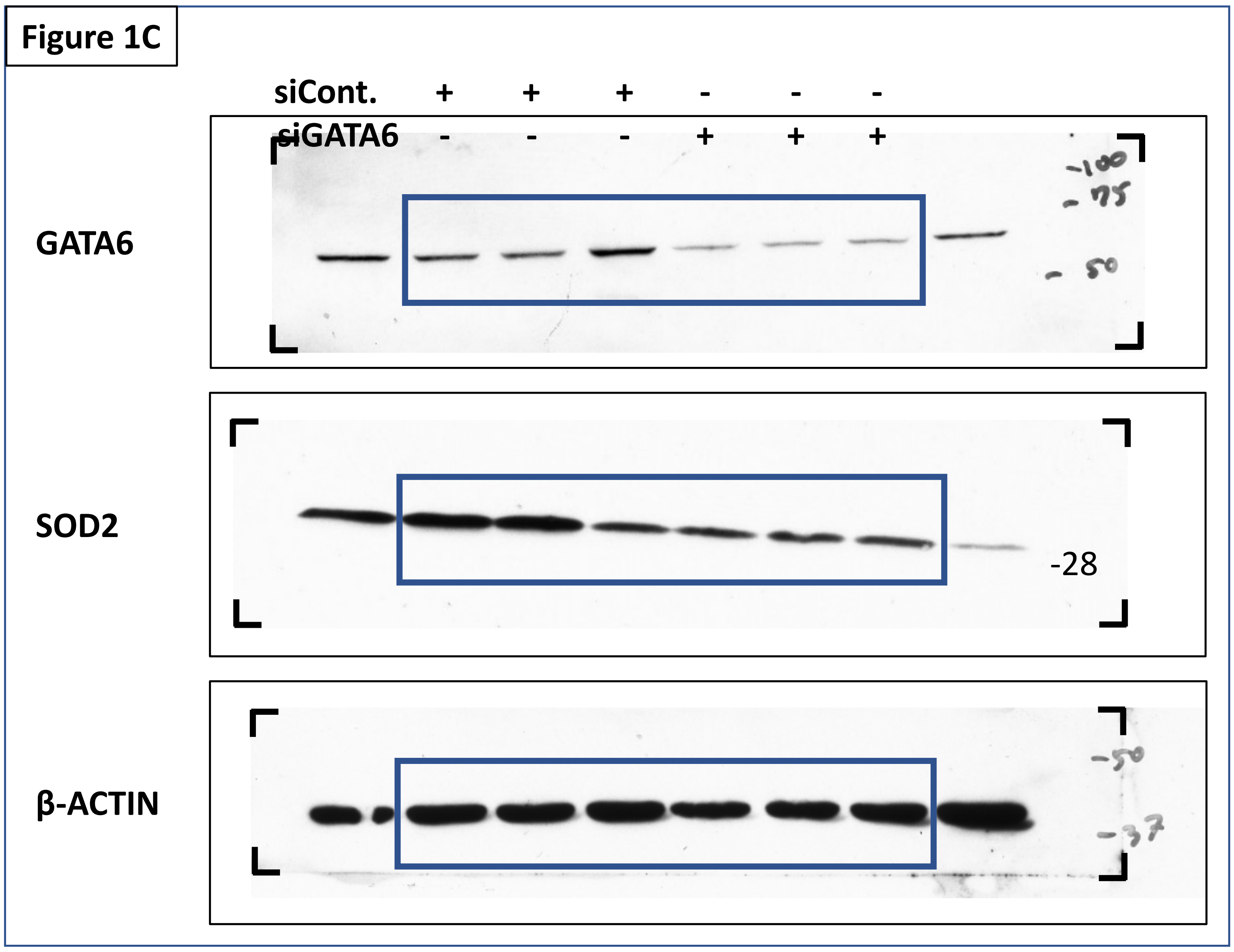

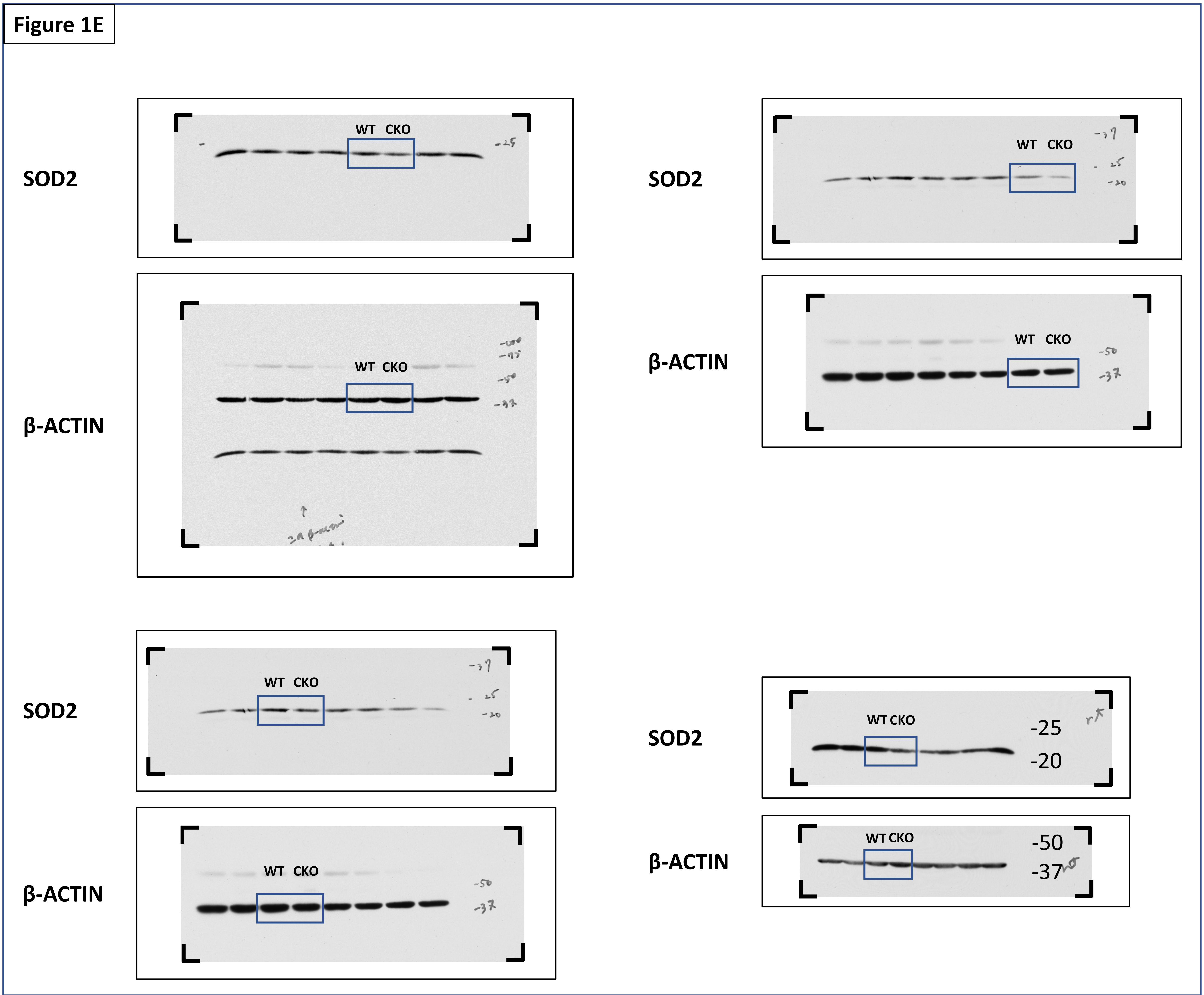

Figure S14. Original immunoblot images for Figure 2.The full length immunoblot for Figures 2D Histone 3 and 2F SOD2 and Tubulin for SOD2 could not be provided because blots were cut prior hybridization with antibodies.

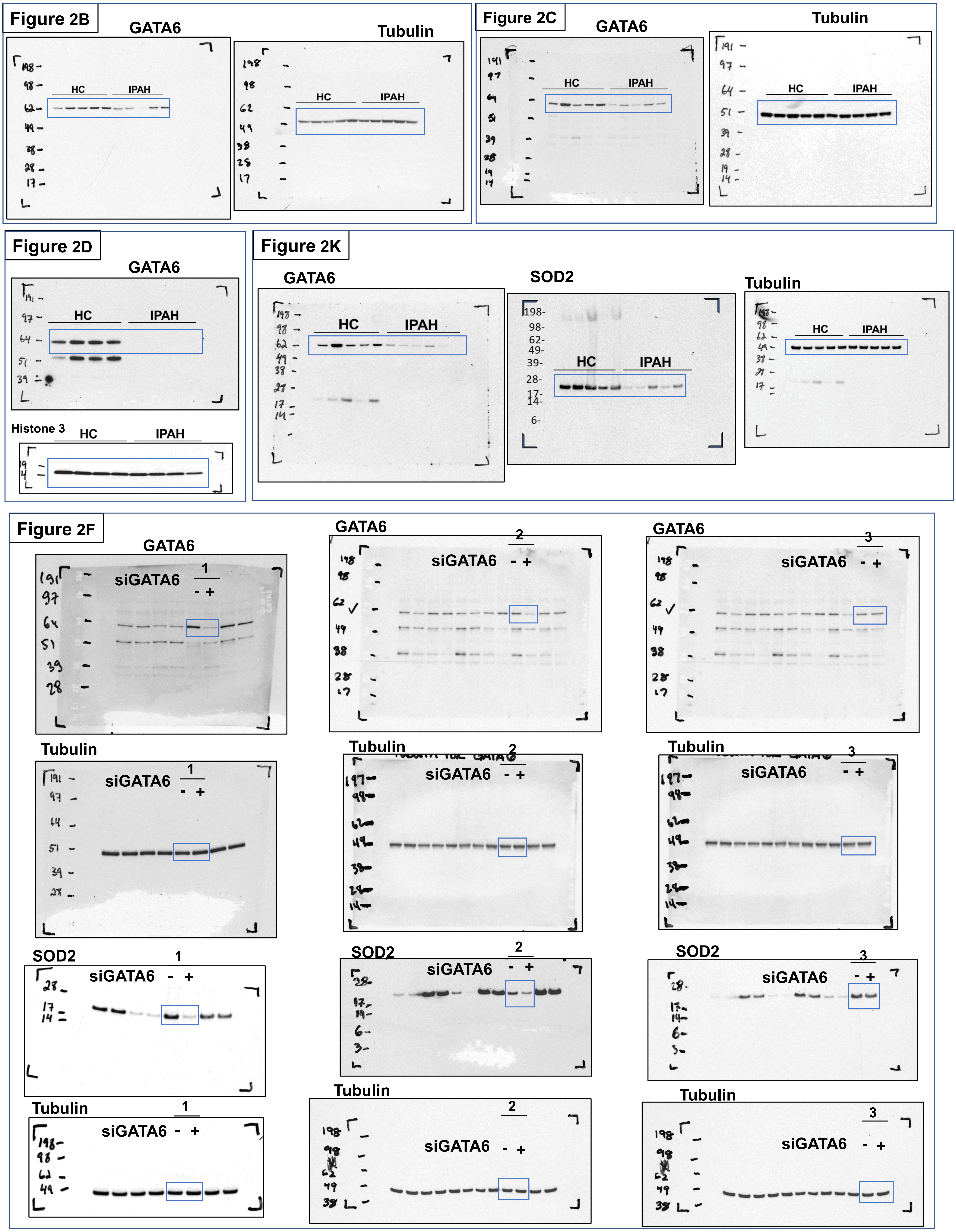

Figure S15a. Original immunoblot images for Figure 3A-3E.

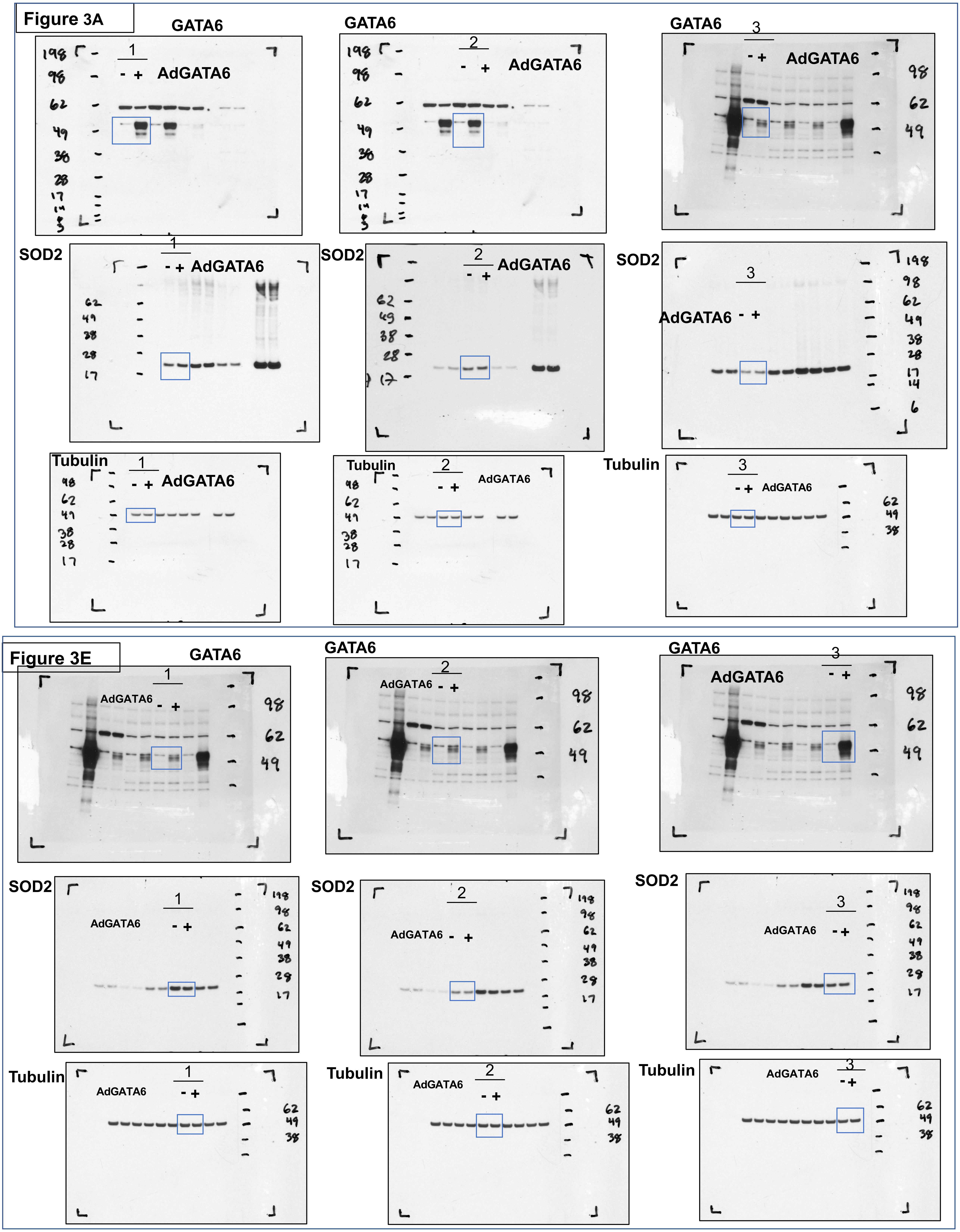

Figure S15b. Original immunoblot images for Figure 3J **GATA6, PRPF4, Tubulin, STING, Tubulin**

Figure 3J : GATA6, PRPF4, Tubulin, STING, Tubulin

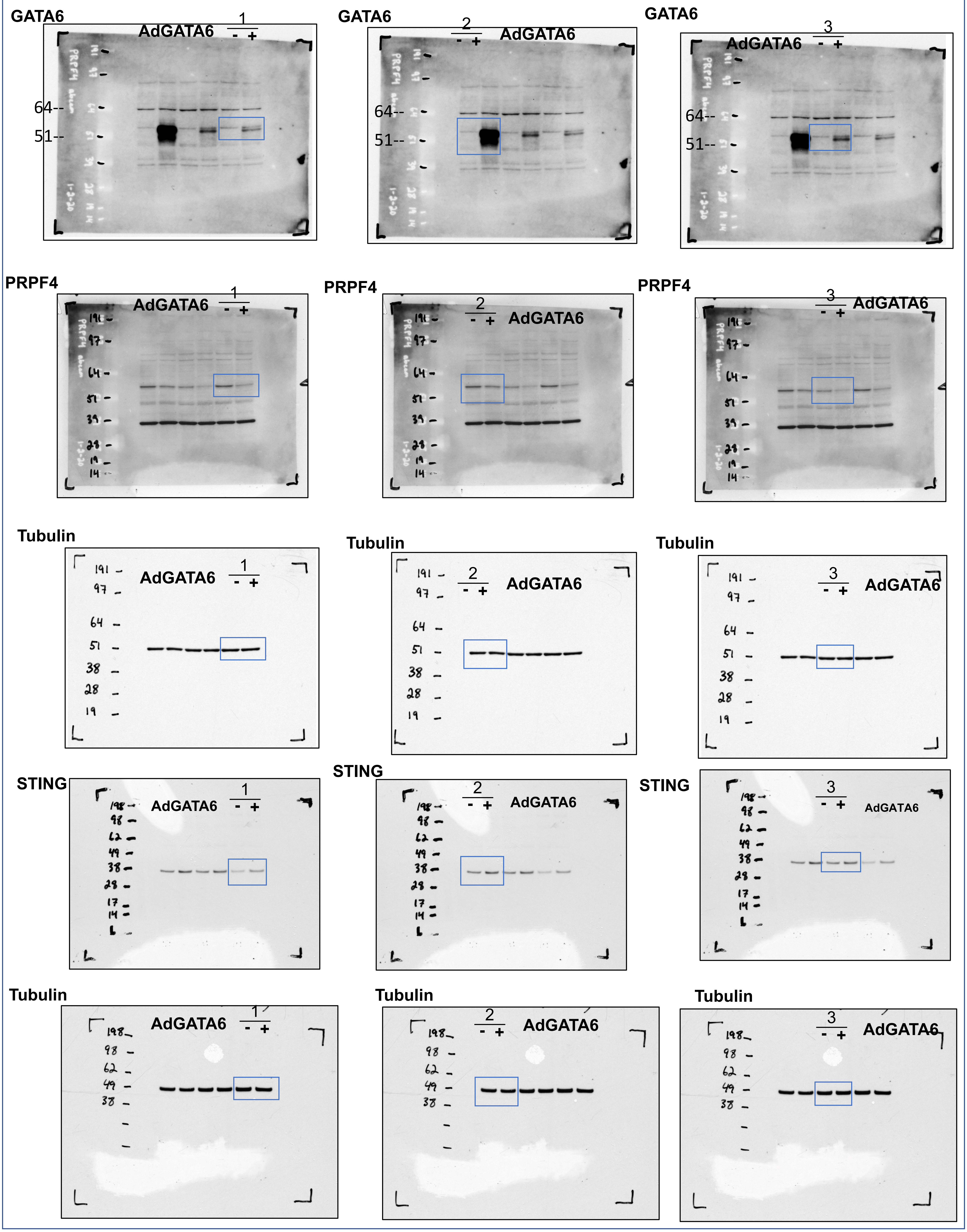

Figure S15c. Original immunoblot images for Figure 3J, 3K

Figure 3J (EAF1, Tubulin, MYEOV, Tubulin)

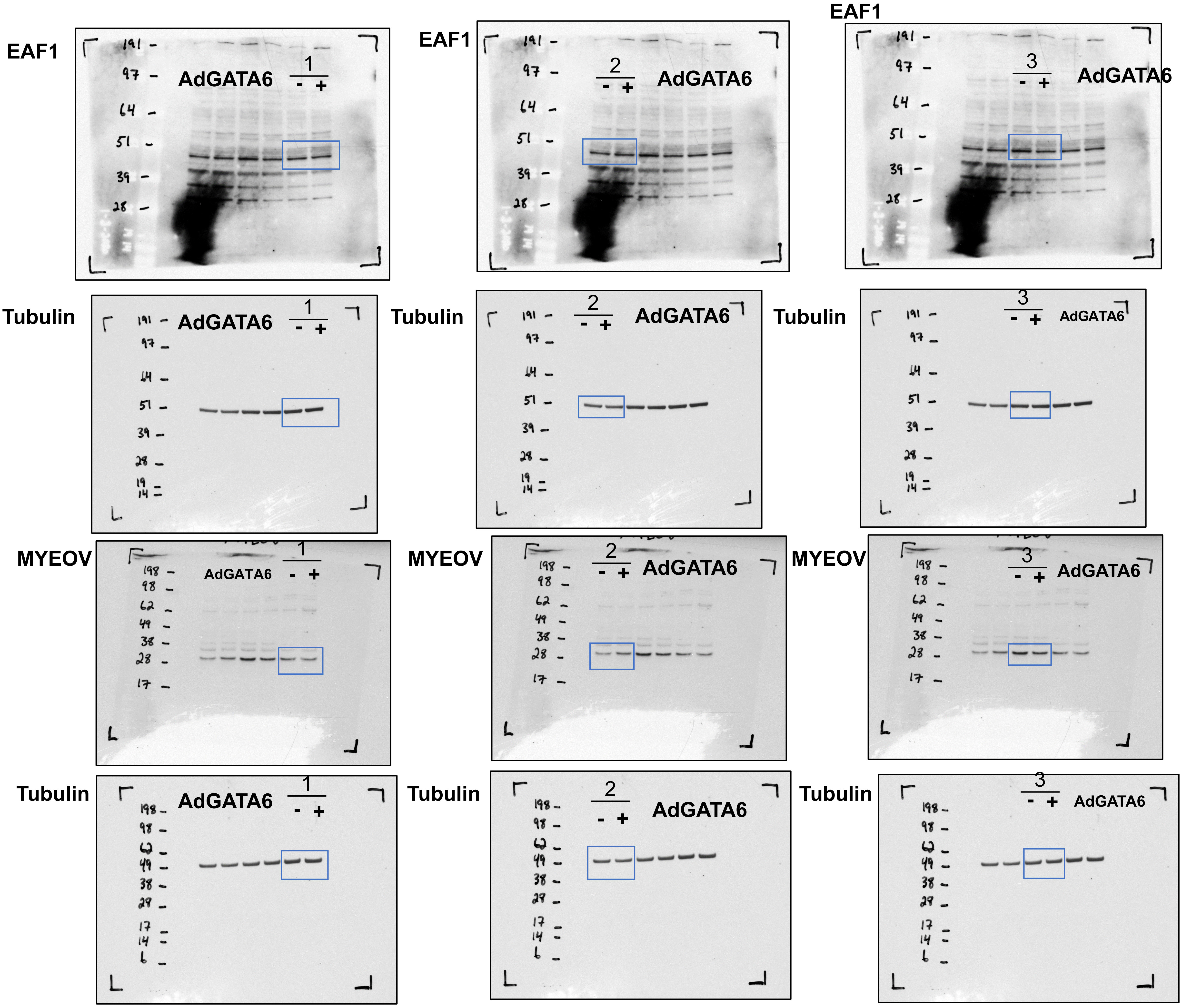

Figure 3K

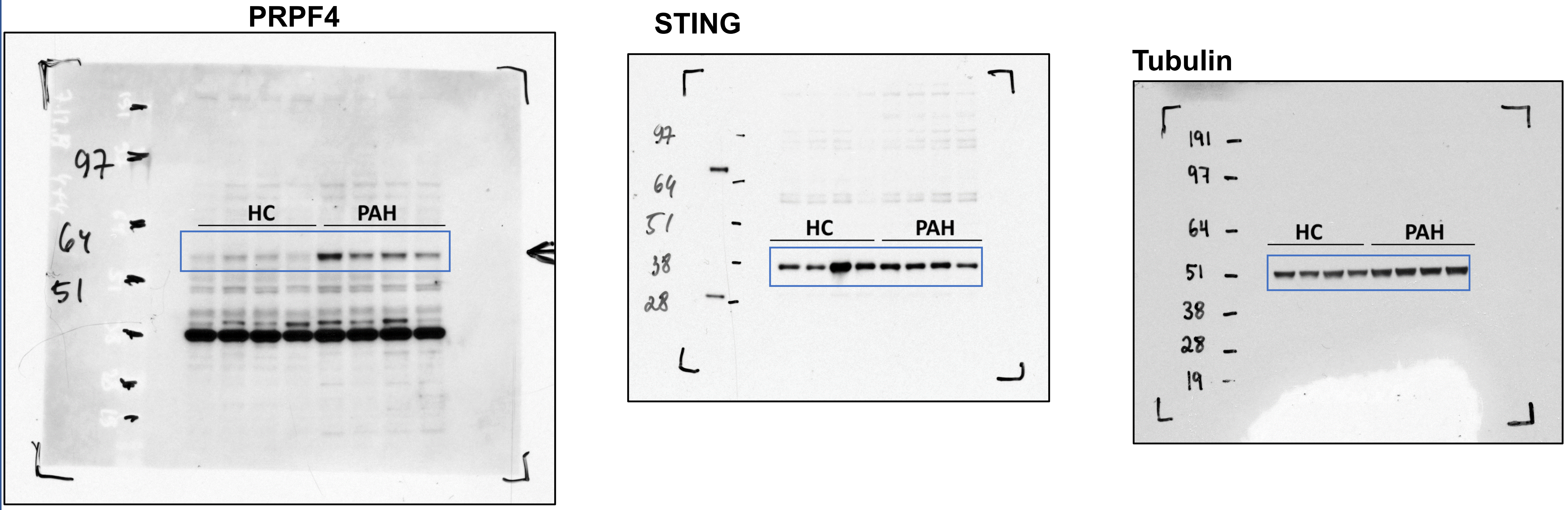

Figure S16a. Original immunoblot images for Figure 4B

The full length immunoblots for Figures 4B could not be provided because blots were cut prior to hybridization with antibodies.

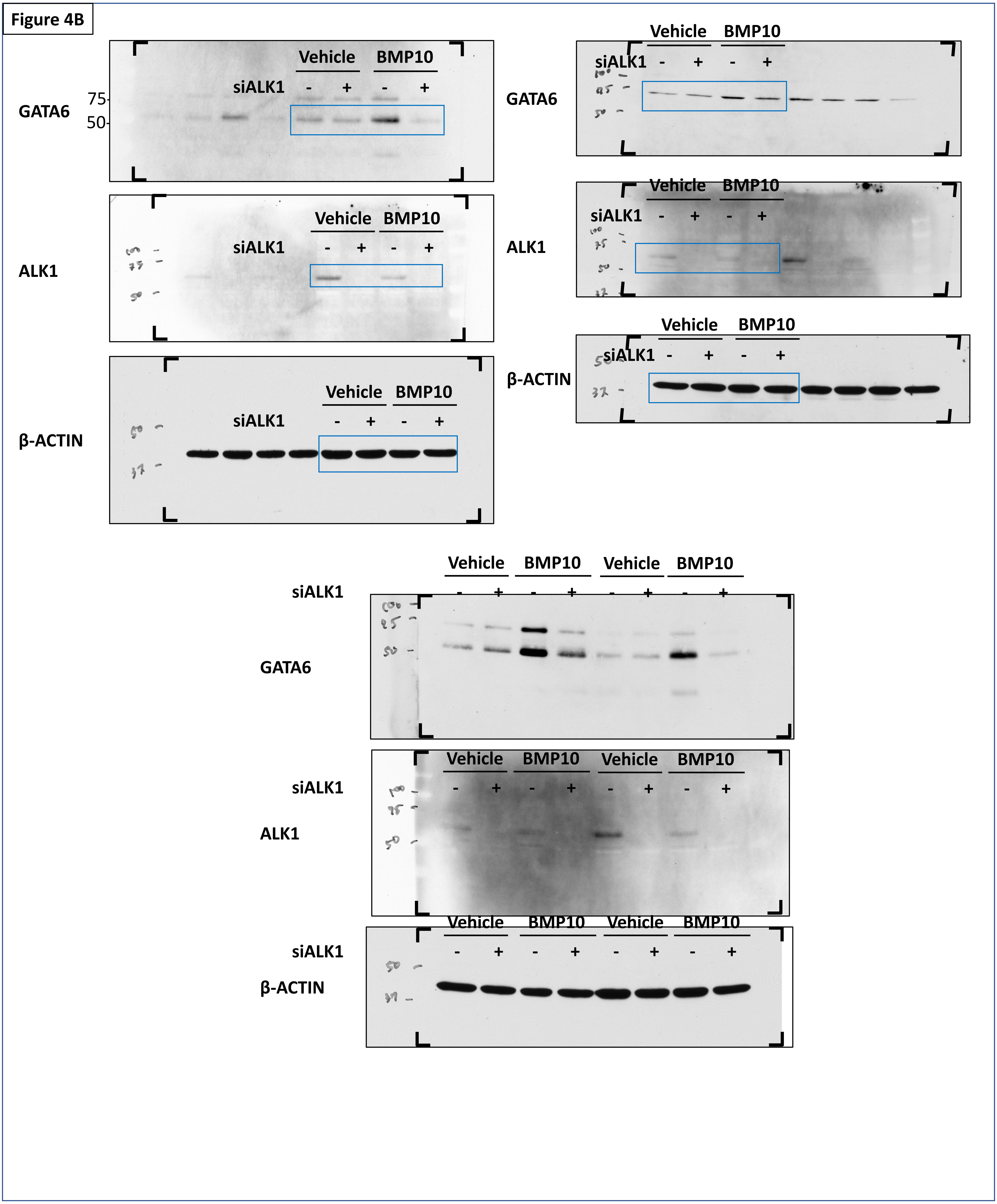

Figure S16b. Original immunoblot images for Figure 4D
All full length immunoblots for Figures 4D could not be provided because some blots were cut prior to hybridization with antibodies.

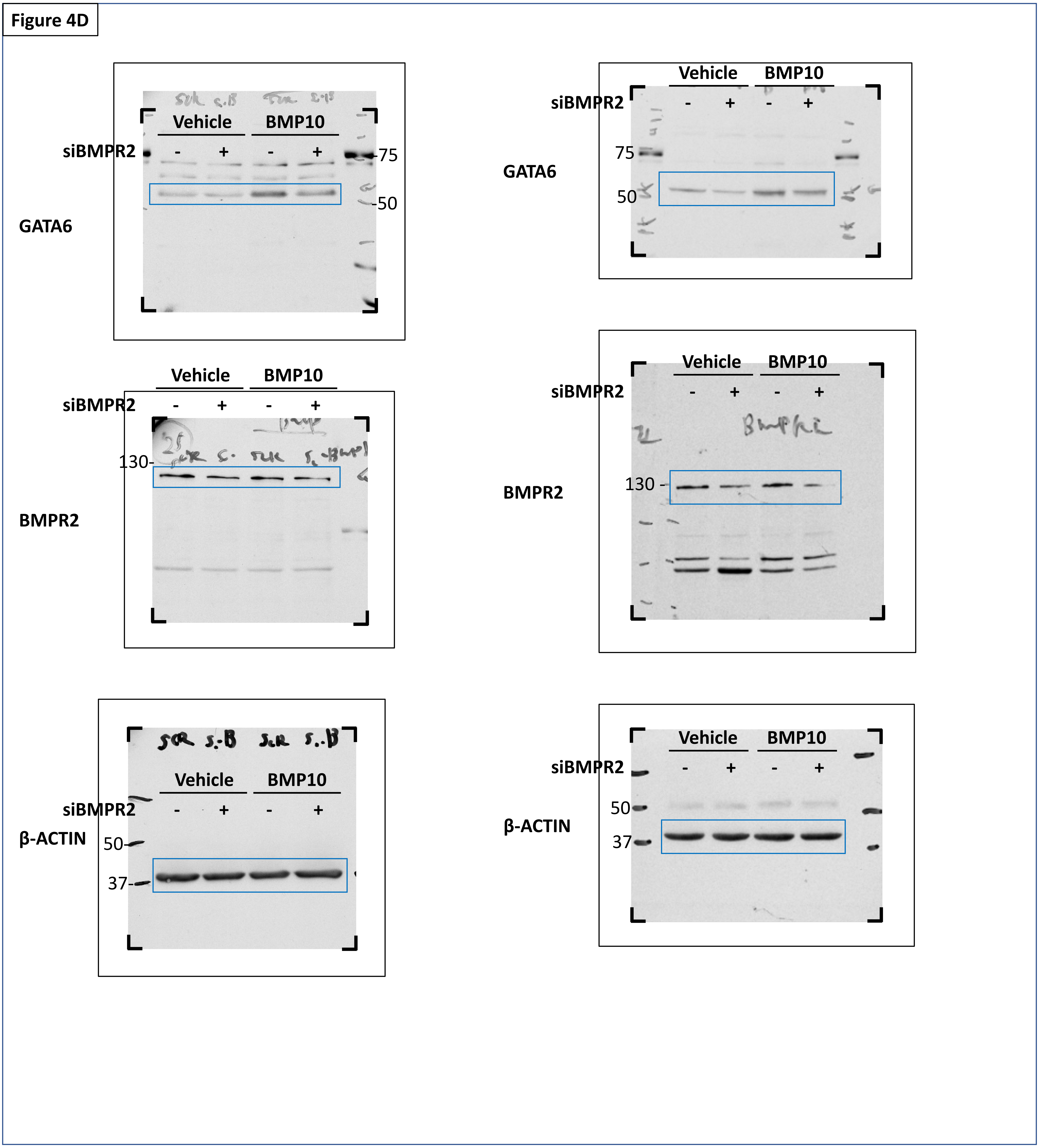

Figure S16c. Original immunoblot images for Figure 4D
All full length immunoblots for Figures 4D could not be provided because some blots were cut prior to hybridization with antibodies.

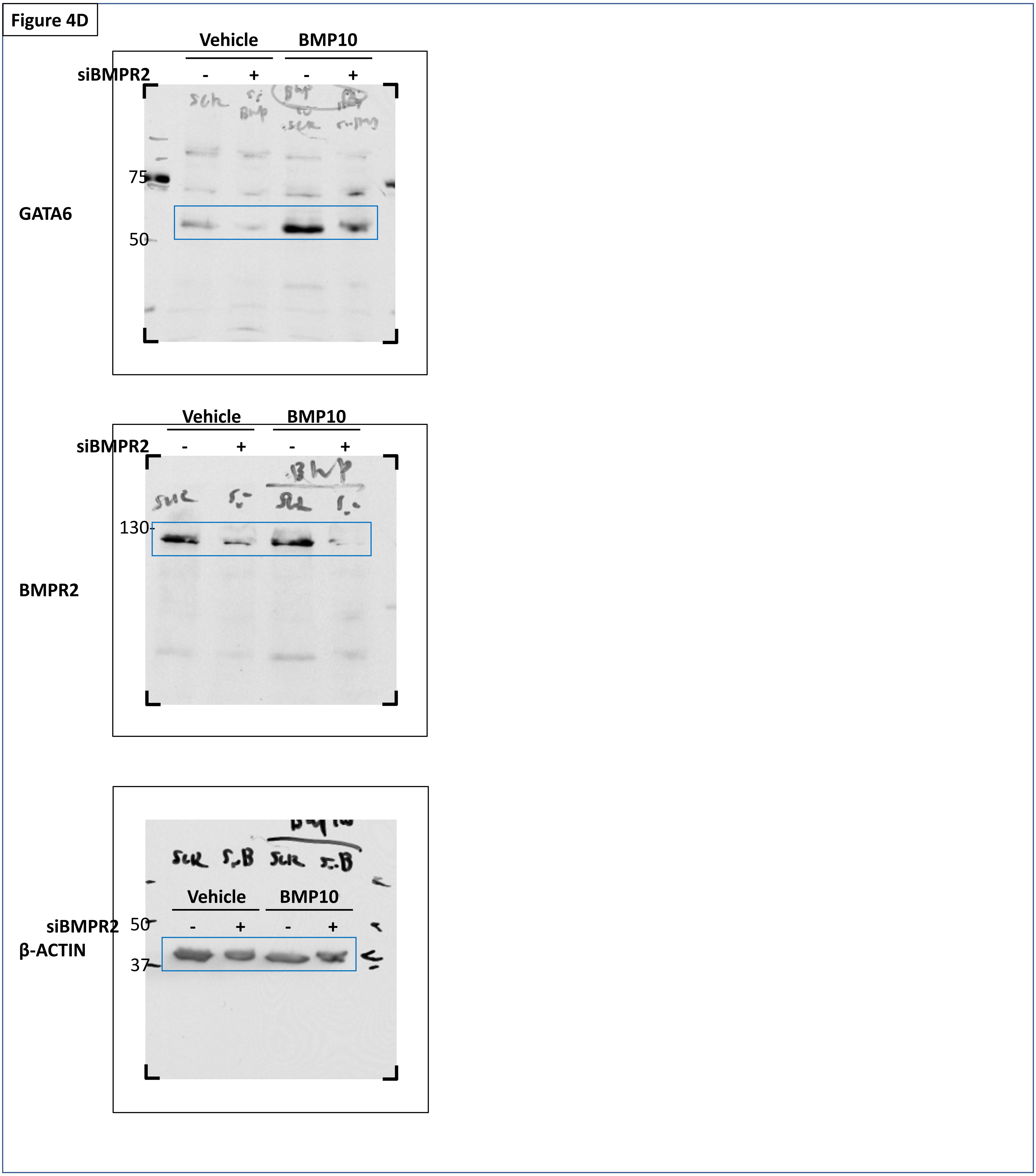

Figure S16d. Original immunoblot images for Figure 4F.
The full length immunoblots for Figure 4F (first set) could not be provided for all westerns as the blot was cut vertically after probing with Endoglin. The right side of the blot is provided, and the left side was used for unrelated experiments.

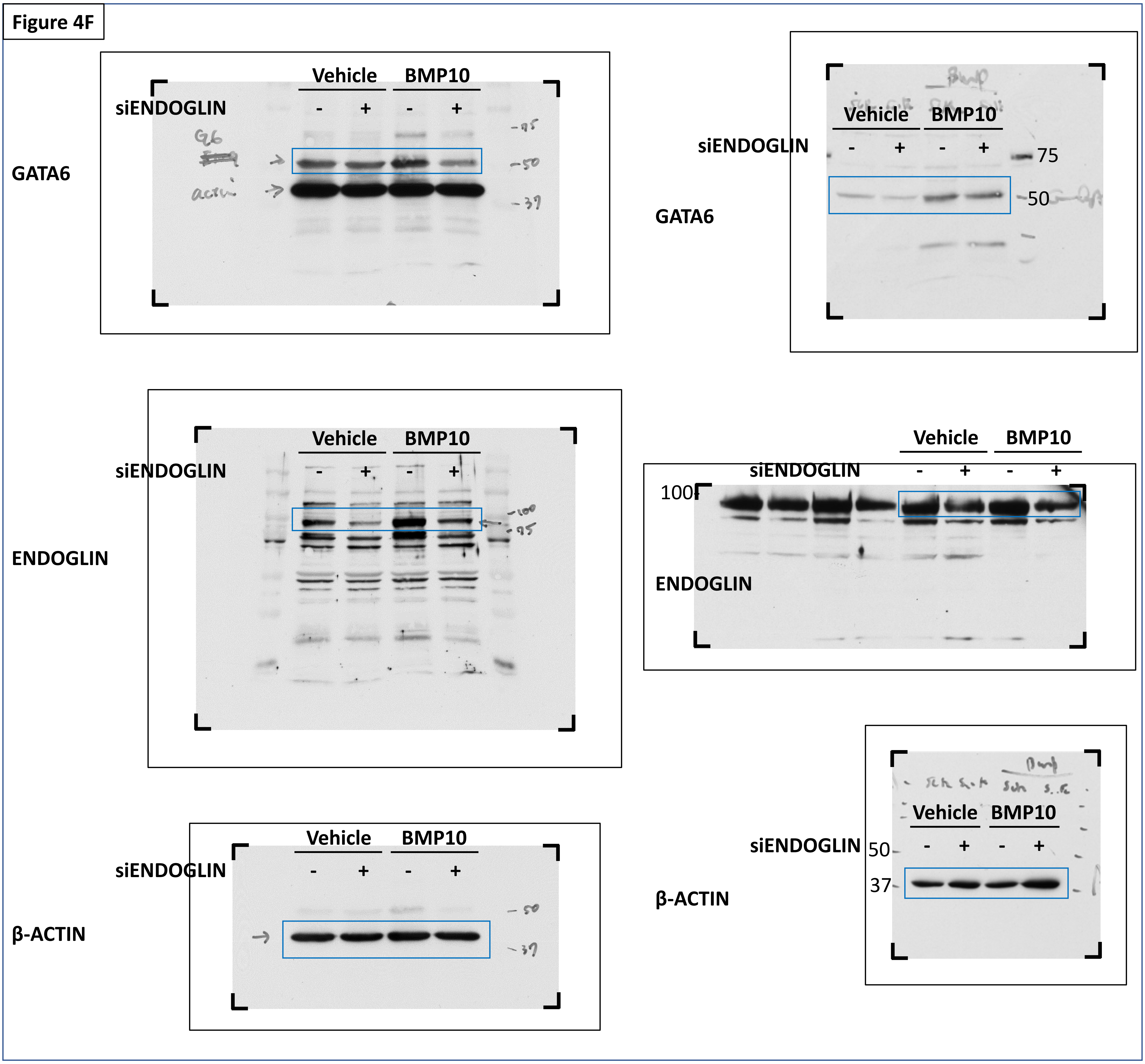

Figure S16e. Original immunoblot images for Figure 4F.
All full length immunoblots for Figures 4F could not be provided because some blots were cut prior to hybridization with antibodies.

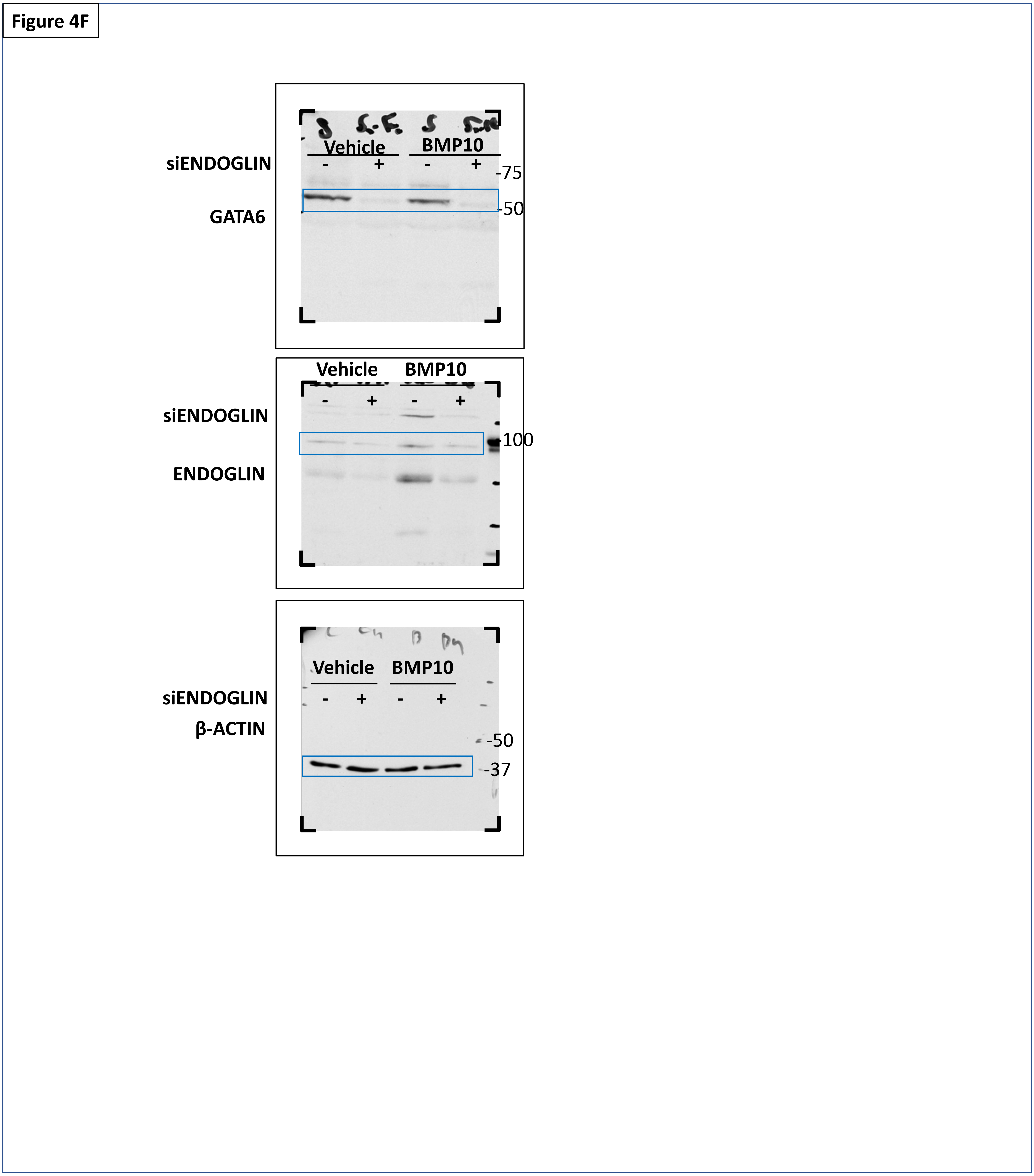

Figure S16f. Original immunoblot images for Figure 4 G,N.

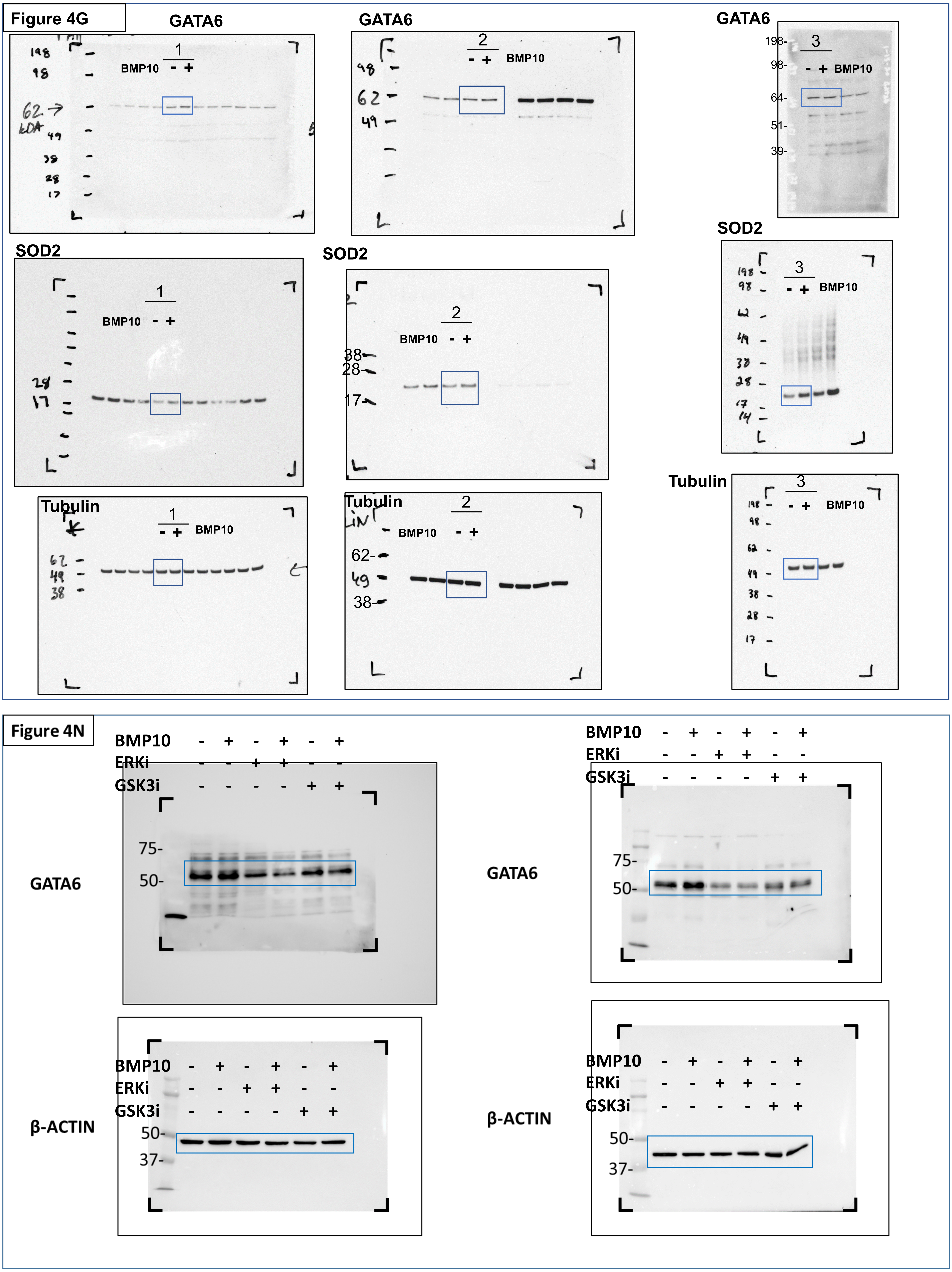

Figure S17a. Original full-length gels for Fig 5B and original immunoblot images for Figure 5C.

Figure 5B

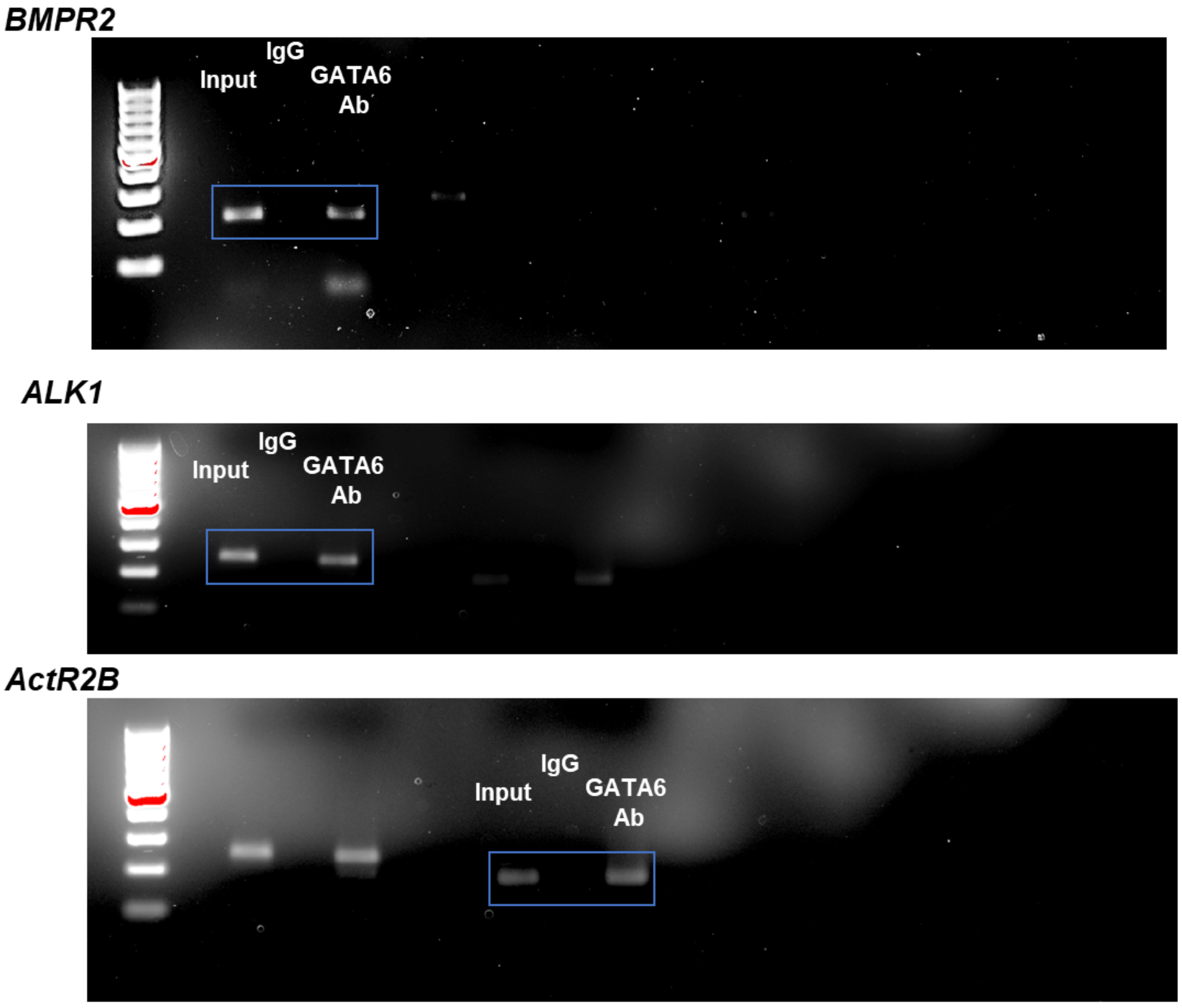

Figure 5C

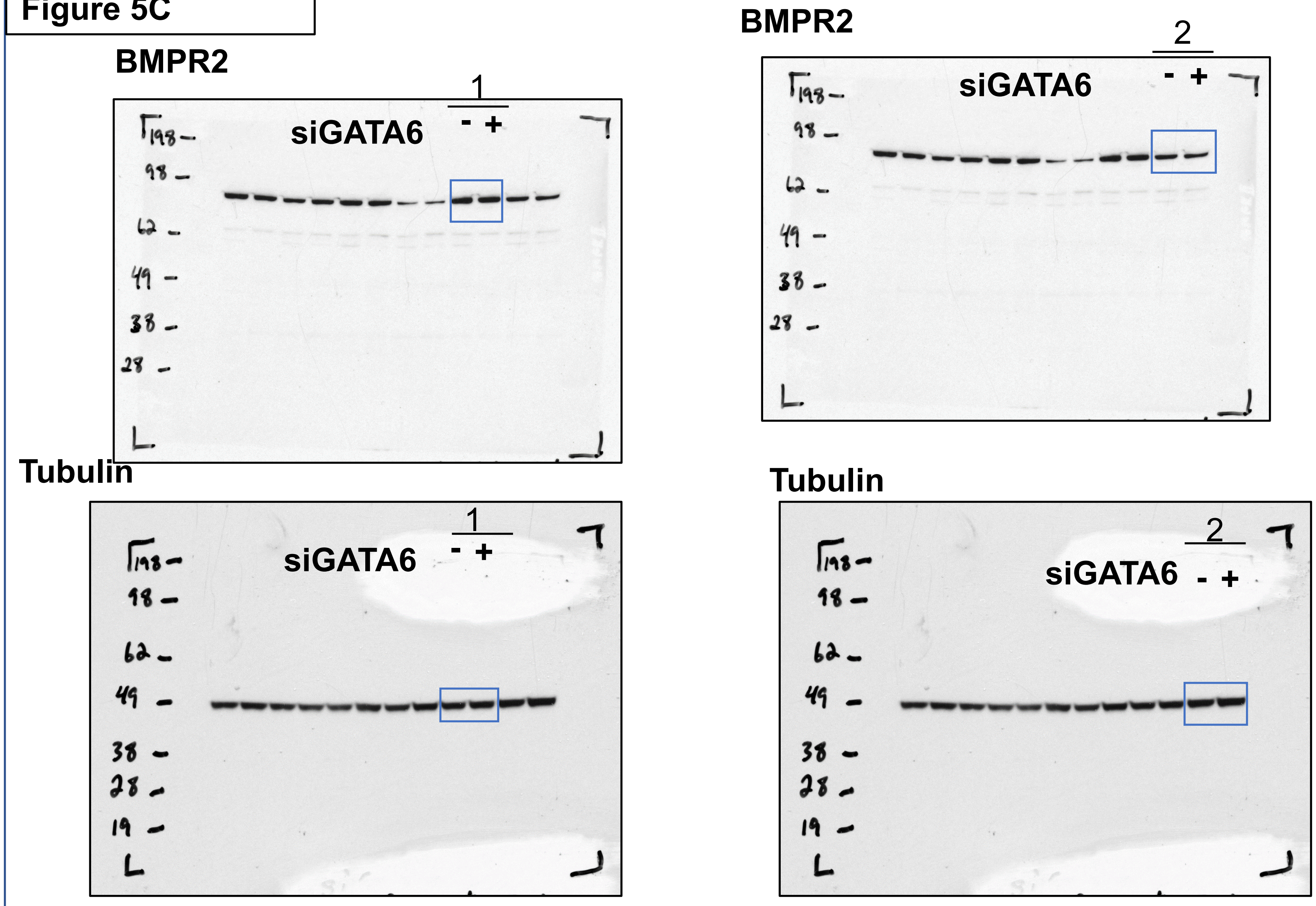

Figure S17b. Original immunoblot images for Figure 5G.
All full length immunoblots for Figures 4D could not be provided because some blots were cut prior to hybridization with antibodies

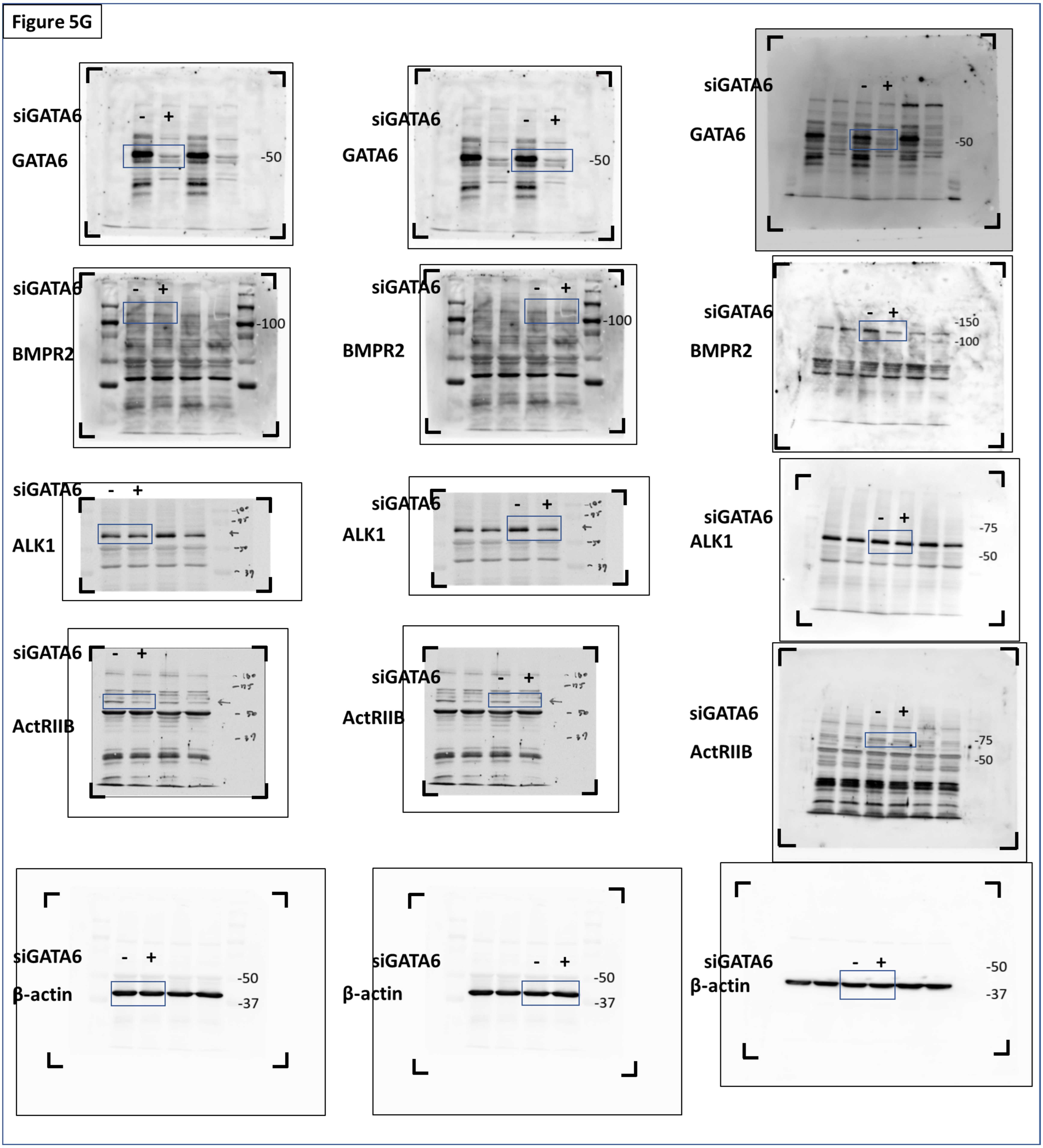

Figure S17c. Original immunoblot images for Figure 5I
The full length immunoblots for some proteins could not be provided because some blots were cut prior to hybridization with antibodies.
.

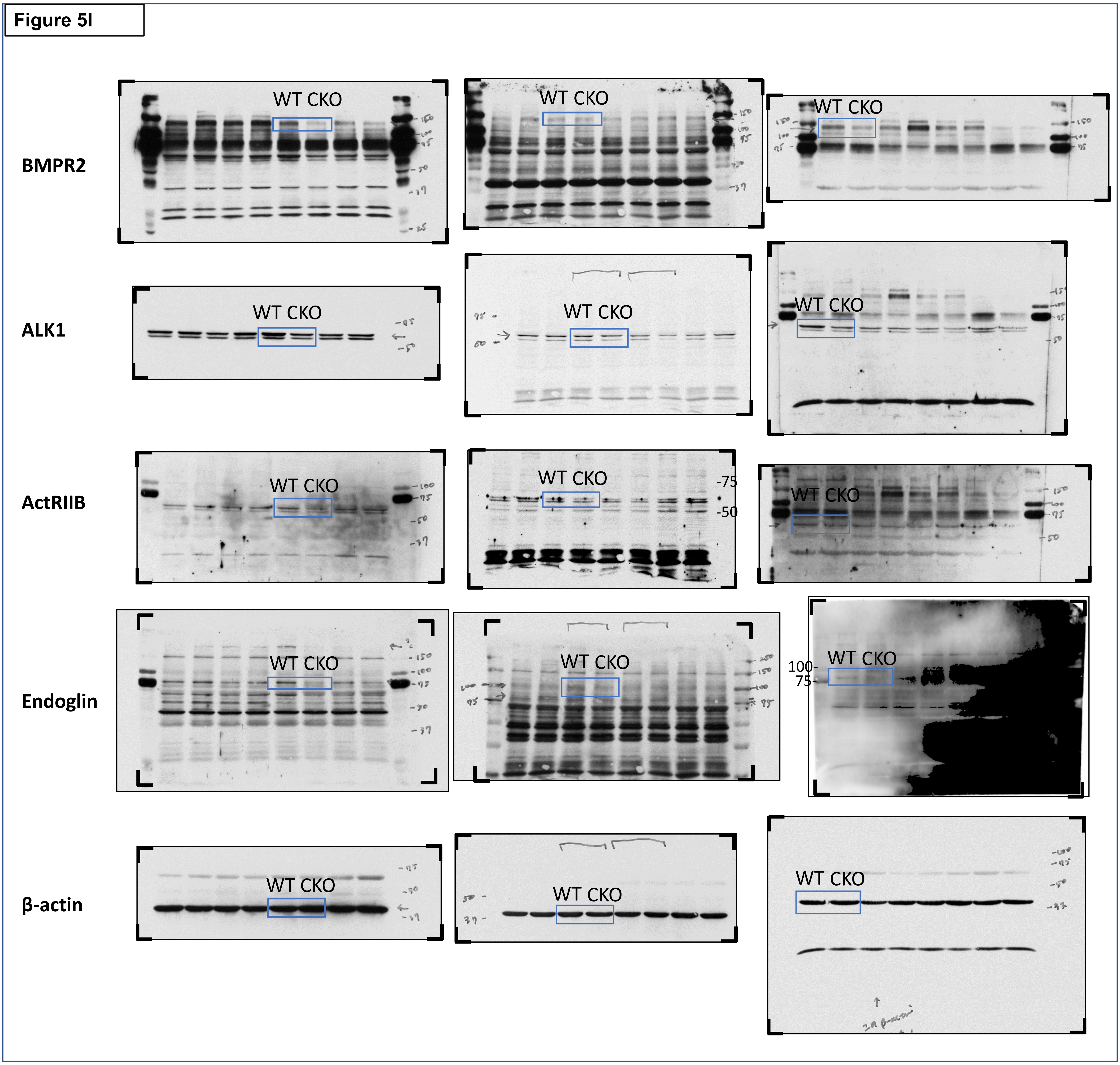

Figure S18. Original immunoblot images for Figure 6

The protein extract for the first blot were hybridized with SOD2, GATA6, BMPR2 . The same extract was then run on a fresh blot and hybridized with BMPR2 and β-actin

.

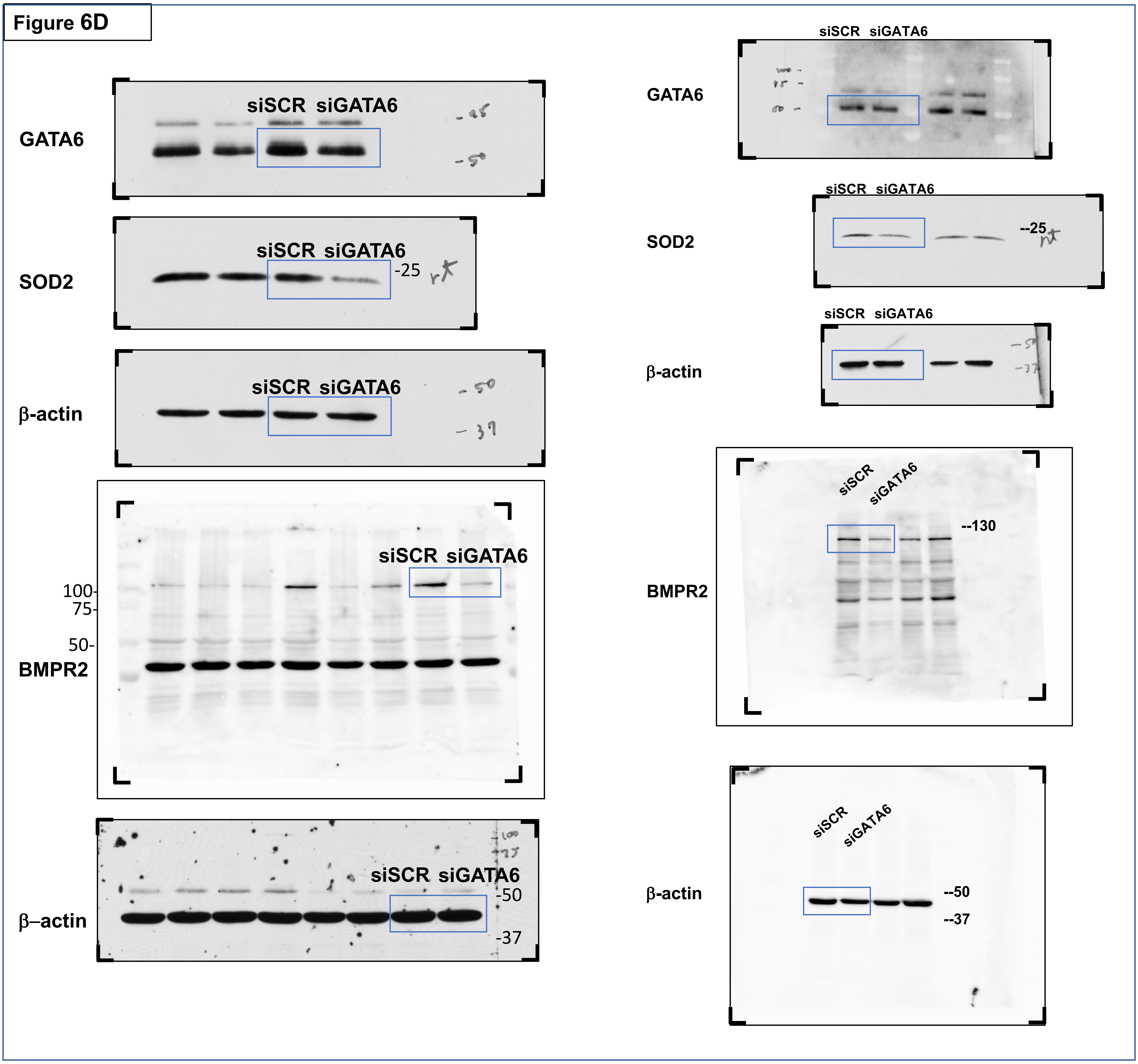

Supplement: Supplementary file 2 — Supplementary Figures. [file 41598_2023_33779_MOESM2_ESM.pdf]
