# Supplementary material for: GATA6 coordinates cross-talk between BMP10 and oxidative stress axis in pulmonary arterial hypertension
Source: Sci Rep. 2023 Apr 22;13:6593. doi: 10.1038/s41598-023-33779-8 (PMC10122657; doi:10.1038/s41598-023-33779-8)
Supplement: Supplementary file 4 — Supplementary Tables. [file 41598_2023_33779_MOESM4_ESM.pdf]

Supplemental Tables

Supplemental Table 1. Gene expression analysis of the antioxidant enzymes in human PAECs (HPAECs) transfected with siGATA6 or control scr siRNA (see excel file "Supplemental Figures 1-3")

Supplemental Table 2. RNA-seq gene expression in PAH PAVSMC infected with sham or GATA6 expressing Adenovirus, filtered for genes showing average expression by GATA6 infected cells>50 (see excel file "Supplemental Figures 1-3)

Supplemental Table 3. Pathway analysis of RNA-seq gene expression in PAH PAVSMC infected with sham or GATA6 expressing Adenovirus (see excel file "Supplemental Figures 1-3)

a. Pathways upregulated by genes associated with GATA-6 overexpression.
b. Pathways downregulated by genes clustering with GATA-6 overexpression

Supplemental Table 4. Human subjects' characteristics

#	Condition	Gender	Age, years	Age, years (Mean ± SE)
1	Non-diseased	F	33	42.2±3.3
2	Non-diseased	F	43	
3	Non-diseased	F	57	
4	Non-diseased	F	64	
5	Non-diseased	F	22	
6	Non-diseased	F	50	
7	Non-diseased	F	53	
8	Non-diseased	F	38	
9	Non-diseased	F	56	
10	Non-diseased	F	57	
11	Non-diseased	F	22	
12	Non-diseased	F	36	
13	Non-diseased	F	71	
14	Non-diseased	F	44	
15	Non-diseased	F	23	
16	Non-diseased	M	24	

17	Non-diseased	M	47	
18	Non-diseased	M	40	
19	Non-diseased	M	41	
20	Non-diseased	M	23	
21	IPAH	F	29	41.2±3.4
22	IPAH	F	32	
23	IPAH	F	16	
24	IPAH	F	36	
25	IPAH	F	40	
26	IPAH	F	50	
27	IPAH	F	62	
28	IPAH	F	49	
29	IPAH	F	40	
30	IPAH	F	16	
31	IPAH	F	36	
32	SSc-PAH	F	53	
33	SSc-PAH	F	52	
34	IPAH	M	21	
35	IPAH	M	53	
36	IPAH	M	51	
37	SSc-PAH	M	63	
38	SSc-PAH	M	42	

F—female; M—male; IPAH—idiopathic pulmonary arterial hypertension; SSc-PAH - systemic sclerosis - pulmonary arterial hypertension.

Supplemental Table 5 Mouse qPCR primers

Gene	Forward primer	Reverse primer
B2MG	TCGCTCGGTGACCCTAGTCTTT	ATGTTCGGCTTCCCATTCTCC
Gata6	CCAGCAGGACCCTTCGAAAC	CGCATGCATTGCACAGGTAAT
Gpx1	TTCGGACACCAGGAGAATGG	TAAAGAGCGGGTGAGCCTTC
Gpx2	TCGGACATCAGGAGAACTGTC	TGCCCATTGACGTCACACTT
Gpx3	TTCCTGAAGAACTCCTGCCCT	CCAGCGGATGTCATGGATCT
Gpx4	CCAAAGTCCTAGGAAACGCCC	GGGATGCACACAAGCCCAG

Gpx5	CCTTCTTGAAGCGTTCTTGTCC	TCCAGCGGATGTCATGGACT
Gpx6	GTCACGGTTTTGGGCTTTCC	GCCTGGACGCACATACTTGA
Gpx7	ACCGTGGCTCGGTTTCCC	CAAACTGGTTGCAAGGGAAGG
Gsr	TGGCACTTGCGTGAATGTTG	TGTTCAGGCGGCTCACATAG
Gss	CACTGGGTCGTACCGAAGC	ATCCCAACTCGCTCGTTGTC
Gstm4	AGTCAAAGTTGCTCACACCG	CGGTCATAGTCAGGAGCGTC
Gstt2	CTTGTGTCGCTACCGGCAAT	CTCATGTGCTGCCCTTTGAGT
Nox1	CTTGCACCGATTGCTTTTTAT	CATTAGATGGGTGCATGACAA
Nox2	TGCCAACTTCCTCAGCTACA	GTGCACAGCAAAGTGATTGG
Nox4	CCCTAAACGTTCTACTTTTCTGGA	TGCTCTGCTTAAACACAATCCT
Sod2	AGGAGAGTTGCTGGAGGCTA	AGCGGAATAAGGCCTGTTGTT
Cat	CCTTCAAGTTGGTTAATGCAGA	CAAGTTTTTGATGCCCTGGT
eNOS	CCAGTGCCCTGCTTCATC	GCAGGGCAAGTTAGGATCAG
Bmpr2	GAGCCCTCCCTTGACCTG	GTATCGACCCCGTCCAATC
Alk1	GCCCTCTAGAGACAACTATCTTCAA	TGTGGTTCCACTCACACACC
ActR2B	TCTCTGGGGATCGCTGTG	GTTGGCGTTGTAGTAGATGCAC

Supplemental Table 6 Human qPCR primers

Gene	Forward primer	Reverse primer
GATA6	TTGTGGACTCTACATGAAACTCCA	TTATGTTCTTAGGTTTTCGTTTCCTG
GPX1	TATCGAGAATGTGGCGTCCC	TCTTGGCGTTCTCCTGATGC
GPX3	CGGATTGGTCACACCCGAG	ACTTCTCTTGTCCCCGGCT
GPX7	CTTTAACGTGCTCGCCTTCC	ATGAGACACTGTAGGTGCGG
NOX4	GCTGACGTTGCATGTTTCAG	CGGGAGGGTGGGTATCTAA

SOD2	GCAAGGAACAACAGGCCTTA	AAGAGCTTAACATACTCAGCATAAC
CAT	CTCCGGAACAACAGCCTTC	ATAGAATGCCCGCACCTG
GSTM4	AGAGTCTGCTACAGCCCTGA	GCCACAGGAAACCTCAAAGC
GSTT2	AATGGCATCCCCTTAGAGC	CTTGAGCGTCGGCAGTTT
BMPR2	CCCGCTCCTACCTCTCCT	CGCAGAACAACCGTGAGAG
ALK1	AGACCCCCACCATCCCTA	CGCATCATCTGAGCTAGGC
ActR2B	TGTCAAGATCTTCCCACTCCA	CATGCCAGGTGTGCTGAA
Endoglin	ACTGCTGCACTCTGGTACATC	AATCCCTCAGAGGCTTCACTG

Supplemental Table 7 ChIP-PCR primers

Primer		Primer sequence	PCR Fragment	Location From TSS (bp)
SOD2	F	TAACTGTGCCCAGGAGAAGC	189	-655 ~ -652
	R	GGACAGGAGGCACACAGACT		
GPX1	F	CAAGGAATTTCTTAACTTCTTGCTTC	156	-1498 ~ -1495
	R	CGGAGTAAAGCCCTGAAGTG		
GPX7	F	GCCCTCCAGGCTAGTTTAGG	199	-749 ~ -746
	R	AAGGGCTAAGCACTCCTGTG		
BMPR2	F	GAATCCGGGGTTGGTATTGC	206	-1359 ~ -1356
	R	TGCTGGTCTTTCATTGTGGC		
ALK1	F	CTCCACCCACCCTTTCCTAG	235	-699 ~ -694
	R	ATCCCAGGTTTCCAGACTCC		
ActR2B	F	CTCCCTTTCAGCTGCTCCT	165	-239 ~ -235
	R	ACTAGAGCTTCGGAGGAACG		
